# Superlative photoelectrochemical properties of 3D MgCr-LDH nanoparticles influencing towards photoinduced water splitting reactions

**DOI:** 10.1038/s41598-022-13457-x

**Published:** 2022-06-03

**Authors:** Susanginee Nayak, Kulamani Parida

**Affiliations:** Centre for Nano Science and Nano Technology, Institute of Technical Education and Research (ITER), Siksha ‘O’ Anusandhan Deemed to Be University, Bhubaneswar, Odisha 751030 India

**Keywords:** Chemistry, Energy science and technology, Materials science, Nanoscience and technology

## Abstract

In the present work, we report the synthesis of single system three-dimensional (3D) open porous structure of MgCr-LDH nanoparticles in a substrate-free path by using one-step formamide assisted hydrothermal reaction followed by visible light irradiation for significant photoelectrochemical (PEC) properties that manifest towards photocatalytic H_2_ and O_2_ production. The as-prepared nanostructured materials were characterized by various physico-chemical characterization techniques. Moreover, this unique synthetic approach produces 3D open porous network structure of MgCr-LDH nanoparticles, which were formed by stacking of numerous 2D nanosheets, for effective light harvestation, easy electronic channelization and unveil superlative PEC properties, including high current density (6.9 mA/cm^2^), small Tafel slope of 82 mV/decade, smallest arc of the Nyquist plot (59.1 Ω cm^−2^) and photostability of 6000 s for boosting water splitting activity. In addition, such perfectly self-stacked 2D nanosheets in 3D MgCr-LDH possess more surface active defect sites as enriched 50% oxygen vacancy resulting a good contact surface within the structure for effective light absorption along with easy electron and hole separation, which facilitates the adsorption of protons and intermediate for water oxidation. Additionally, the Cr^3+^ as dopant pull up the electrons from water oxidation intermediates, thereby displaying superior photocatalytic H_2_ and O_2_ production activity of 1315 μmol/h and 579 μmol/h, respectively. Therefore, the open 3D morphological aspects of MgCr-LDH nanoparticles with porous network structure and high surface area possess more surface defect sites for electron channelization and identified as distinct novel features of this kind of materials for triggering significant PEC properties, along with robustly enhance the photocatalytic water splitting performances.

## Introduction

In progress of time, the rapid and massive exhaustion of traditional fuels, such as crude petroleum oil, coal, and natural gas, accelerates the high demand in the advancement of sustainable energy resources to meet up the adequate requirements^1^, and it is quite imperative to enlarge the green and clean energy sources such as solar energy, H_2_ energy, hydrothermal energy, wind energy, tidal energy, and geothermal energy, etc., to lessen the environmental majors^2–4^. Opportunely, photocatalytic (PC) or PEC water splitting to produce H_2_ and O_2_ is considered as one of the most superfluities of green technological approaches to indulgence the solar-to-chemical energy conversion for addressing the worldwide energy shortage^5,6^. The hydrogen evolution reaction (HER), and oxygen evolution reaction (OER), has been investigated for decades and regarded as vital energy conversion reaction as in water electrolysers^7,8^. However, the slow OER kinetics, including the 4 electron transfer to form O_2_ requires a potentate catalyst, which could minimize the overpotentials required in water splitting ^9,10^. Since from the foremost invention of PEC water splitting on TiO_2_ photoelectrode by Fujishima and Honda^11^, diverse low-cost, abundant and renewable photocatalytic materials such as Bi_2_WO_6_^12,13^_,_ g-C_3_N_4_^14–18^, CdS^19,20^, MoS_2_^21,22^, graphene^22–24^, MXene^25–27^, graphdiyne^28^, etc., have been developed for their sustainability, suitability and efficiency for water splitting reactions. In addition, recently CoWO_4_ nanocubes^29^, CuWO_4_ nanoparticles (NP)^30^, α-Fe_2_O_3_@g-C_3_N_4_^31^, Ni/MoO_2_@N-doped-carbon^32^, NiMoO_4_-nanorods@rGO^33^, and CuWO_4_@rGO^34^, electrode materials have been developed, which could be regarded as highly effective in water electrolysis reaction^35^.

Especially, layered double hydroxides (LDHs), represented by their molecular formula of [M^2+^_1−x_ M^3+^_x_(OH)_2_] [A^n−^ x/n·mH_2_O], where M^2+^ and M^3+^ represents divalent and trivalent metal cations, and A^n−^ is an anion, regarded as multifaceted 2D layered positively charged nano-photo/electrocatalysts, which have triggered extensive consideration owing to their unique structure, and exceptional properties such as tunable band gap, tailored compositions and availability of suitable surface area leading to a varied range of possible LDH nanostructures for PC and PEC application^36–44^. For illustration, the author, Nayak and Parida et al. has modified NiFe-LDH^16,17,21,45,46^, MgAl-LDH^47,48^, MgCr-LDH^49,50^, based nanohybrid for significant application in PC water splitting along with environmental pollutant abatement purposes. Nevertheless, pristine (PS) LDHs generally manifests feeble quantum efficiency in the presence of solar irradiations as a result of sluggish carrier charge mobility and speedy recombination of excitonic charge pairs that is linked with the deficit of suitable band structure, light absorption tendency, which question about their effectiveness in becoming a robust photocatalyst^51^. Mostly, less exposed reactive sites are associated with the interlayer of pristine LDH for fast adsorption of reactive intermediates and transfer of electrons to reach at the reaction site to promote water splitting. Another subject is that pristine LDHs might undergoes self-degradation of surfaces reactive sites under oxidative conditions^52,53^. Accordingly, novel kind of materials, offering rich reactive sites, fast adsorption of reactive intermediates on defect sites, which draw electrons from intermediates for fast charge separation and provide significant stability throughout the reaction, would symbolize as advancement in water splitting reactions^54,55^. In this regard, numerous strategies has been developed so far accounting lattice doping of metal cation^56^, introducing guest entities into the interlayer^57^, and heterostructure nanohybrid formation of LDH with other semiconductor photocatalysts^16,17,21,45^. Though, these advanced nanohybrid materials displayed outstanding photocatalytic activities as verified in ZnCr-LDH^51^, NiAl-LDH^58^, CoAl-LDH^59^, NiFe-LDH^16^, NiCo-LDH^60,61^, CoFe-LDH^62^, but there is certain room for development in terms of insufficient interaction among counter photocatalysts, undesired active site coverage or blockage, meager excitonic isolation, reduced recyclability etc. In recent times researchers are looking for a way to overcome these hurdles of nanohybrid photocatalytic nanomaterials has put an immense demand for single component LDH based photocatalysts.

Alternatively, an intriguing feature of LDH material is their inherent properties of exfoliation into critical anisotropic 2D nanocrystalline structure of uni/multi-lamellar nanosheets (NS), which composed of ~ 1–10 stacked layers controlled by their synthesis methods and unusual structural features that could be used as building blocks for diverse functional materials^63^. Importantly, during the exfoliation of pristine LDH to nanosheets, oxygen vacancies might be generated which induces the formation of low coordination metallocentres that causes the formation of active catalytic sites of metal oxides/hydroxides/oxyhydroxides for triggering the water splitting reactions^54,55^. It is further noted that numerous exfoliated LDHs having divalent metal cations and abundant metalloactive sites, such as MgCr-LDH^50^, ZnCr-LDH ^64^, CoFe-LDH^65^, NiTi-LDH^66^, and TbZnCr-LDH^67^, have been researched to refine the PEC properties and photocatalytic performances. Besides other exfoliated LDH systems have been established but rarely explored their performances such as MgAl-LDH nanosheets^68^, CoAl-LDH^69^, etc. In these aspects, combination of Mg^2+^ cation with insertion of Cr^3+^ cation as dopant in MgCr-LDHs are significant as Cr^3+^ ions in partial substitution to the octahedral sites of Mg^2+^ cation layers could behave as reactive sites to promote water splitting, while Mg ions offer structural stability of LDHs. In addition, the incorporation of doped Cr^3+^ metal cation into the lattice of MgCr-LDH hold an electronic configuration of t_2g_^3^e_g_^0^, in which the vacant e_g_ orbital could be favorable for the capture of electron from the defect sites and corresponding carrier charge transfer process stabilized the system by enhancing the kinetics of water splitting reactions^70^.

Motivated by the promising properties of exfoliated LDH NS, in this context, we fabricated single system 3D hierarchal porous MgCr-LDH nanoparticles (NP) by assembling and self-stacking of 2D nanosheets via mild hydrothermal strategy followed by visible light irradiation, which established these materials as highly active photocatalyst towards photocatalytic water splitting with enhanced PEC properties for future PEC photoanode materials. These 3D binary MgCr-LDH/NP possess oxygen vacancy (Ov) type defect surface sites, and provides many advantages in variety of ways: (i) superior electronic transportation due to augmentation of the synergic effects amongst Mg^2+^, and Cr^3+^ cations; (ii) Cr^3+^ as dopant, behaves as electron pooler by pulling electrons from the oxygen vacancy sites, which are being used to traps electrons; thus swiftly regenerate the active sites for effective water splitting reactions; (iii) Cr^3+^ as dopant presents in the MgCr-LDH reveals its special electronic configuration of vacant e_g_ orbitals, which facilitates the electronic charge transfer process, thus anticipated to augment the conductivity of Mg(OH)_2_ and certainly promote the photocatalytic water splitting performances and reusability of the MgCr-LDH photocatalyst. Hence, the fabricated 3D open porous structure of MgCr-LDH/NP with high surface area provides more surface active sites relating to oxygen vacancies, and Cr^3+^ as dopant for effective light harvestation, easy electronic channelization, minimum excitonic recombination, excellent stability, etc*.* that eventually leads to significant enhancement in the PEC properties together with promoting photocatalytic H_2_ and O_2_ production rate.

## Results and discussion

### Corroboration of perception

**Figure 1:**
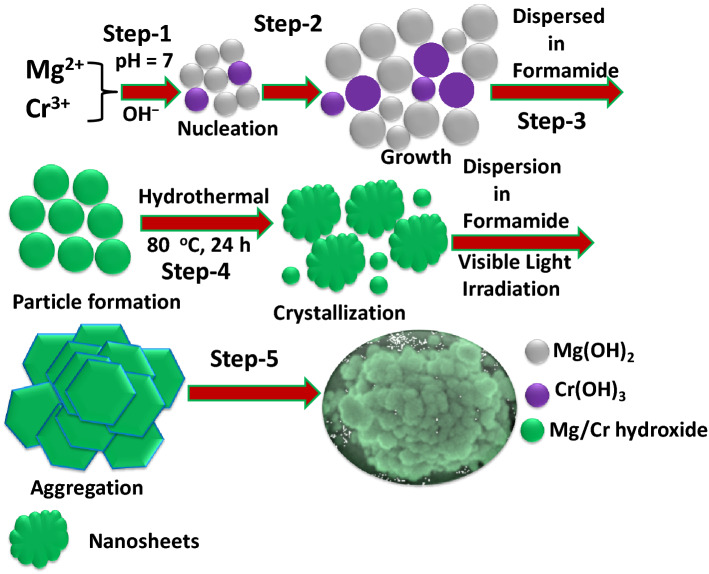
Growth mechanism of MgCr-LDH/NP.

Engineering the morphological features to refrain the existing active sites with creation of new defect sites plays an utmost vital role for an effective excitonic partition and electronic channelization in light driven catalytic reactions^71^. Apart this, the development of green and cost-effective photocatalytic system in terms of substrate-free particulate 3D binary MgCr-LDH/NP via combination of simplistic hydrothermal technique followed by visible light illumination could be regarded as a novel approach towards sustainable energy utilization^72^. This type of 3D binary MgCr-LDH/NP assimilated by 2D nanosheets assured advantages without complex pre/post-treatments together with an effective amalgamation of pre-existing active sites of Cr(OH)_3_ and oxygen vacancy related defect sites for effective electronic transportation resulting magnificent PEC properties towards photoinduced water splitting reactions^73,74^. This resourceful practice certifies single-step synthesis of colloidal MgCr-LDH/NS and thanks to the oxygen vacancies on the MgCr-LDH/NS which mostly provided active sites for further nucleation and crystallization process. The growth process of the 3D MgCr-LDH/NP structures could be described as shown in Fig. 1; a significant and time-saving methodology has been adopted to deliver the significant structural transformation of exfoliated MgCr-LDH/NS to hierarchal 3D structure of MgCr-LDH/NP matrix. Firstly, the well-controlled growth of MgCr-LDH/NS from MgCr-LDH/PS was accomplished by the use of hydrolyzing agent HCHO^75^, together with the OH¯ by using coprecipitation method and dispersion through sonication process^76^. Mostly, hydrolysis of HCHO liberates solvation energy^77^, which prepared the mixed solution of Mg(NO_3_)_2_·6H_2_O and Cr(NO_3_)_3_·9H_2_O, more alkaline and triggers nucleation and growth of MgCr-LDH/NS owing to restricted access to nutrients in confined area. At some point in the reaction process, HCHO acts as ligand binding the Mg^2+^ and Cr^3+^ cations to produce metal complexes in aqueous medium through H-bonding; and causes the complex configuration of polyoxometalate cluster. Thirdly, at mild hydrothermal process of 80 °C, these metal cluster complex shape into 1D sequence using hydrolysis reaction; and chain segments are united to form supramolecular units. Under such instance, the nucleation and growth process of LDH in the successive reaction with OH^−^ and HCHO could hinder so causes the formation of MgCr-LDH/NS. With continuous heating at 80 °C for 12 h, the incremental thickness of the interconnected NS crystallizes into fully-fledged NS. However, after a visible light irradiation of about 30 min, the exfoliated NS entangled and folded to self-stack and cluster shaped into 3D NP. However, the transition state of the MgCr-LDH/NS happened through an unusual route, with advancement of porous 3D MgCr-LDH medium. The morphological alteration started from 2D NS to 3D NP formed by aggregation, self-assembly, and Ostwald process^72^. Further decomposition of HCHO releases formate together with slow liberation of NH_3,_ CO_2_, H_2_ and H_2_O in a restrained gap^50^, but ensure for the porosity and floppiness in the material. In the interim, several H_2_O molecules also penetrate into the interlayer^49,50^. The main reaction steps of MgCr-LDH/NS to MgCr-LDH/NP transformation are given below.

1 $$ {\text{H - CHO }} + {\text{Mg }}\left( {{\text{NO}}_{3} } \right)_{2} \cdot 6{\text{H}}_{2} {\text{O }} + {\text{ Cr}}\left( {{\text{NO}}_{3} } \right)_{3} \cdot 9{\text{H}}_{2} {\text{O }} \to {\text{Ageing}}\,{\text{Mg}}^{2 + } \cdot {\text{Cr}}^{3 + } \left[ {{\text{HCOO}}\left( {{\text{OH}}} \right)} \right] \cdot 13{\text{H}}_{2} {\text{O,}} $$

2 $$ {\text{Mg}}^{2 + } \cdot {\text{Cr}}^{3 + } \left[ {{\text{HCOO}}\left( {{\text{OH}}} \right)} \right] \cdot 13{\text{ H}}_{2} {\text{O }} + {\text{ OH}}^{ - } \to {\text{ Hydrolysis}} \,{\text{Mg }}\left( {{\text{OH}}} \right)_{2} \cdot {\text{Cr}}\left( {{\text{OH}}} \right)_{3} \downarrow + {\text{HCOO}}^{ - } , $$

3 $$ {\text{Mg}}\left( {{\text{OH}}} \right)_{2} \cdot {\text{Cr}}\left( {{\text{OH}}} \right)_{3} \downarrow + {\text{HCOO}}^{ - } \, {\text{In}}\,{\text{hydrothermal }} + {\text{ Light}}\,{\text{irradiation}}\,{\text{MgCr}} - {\text{LDH}}/{\text{NP}}\left( {\text{s}} \right) \, + {\text{ CO}}_{2} \uparrow + {\text{ NH}}_{3} \uparrow + {\text{ H}}_{2} {\text{O}} \uparrow . $$

### Morphological features analysis

**Figure 2:**
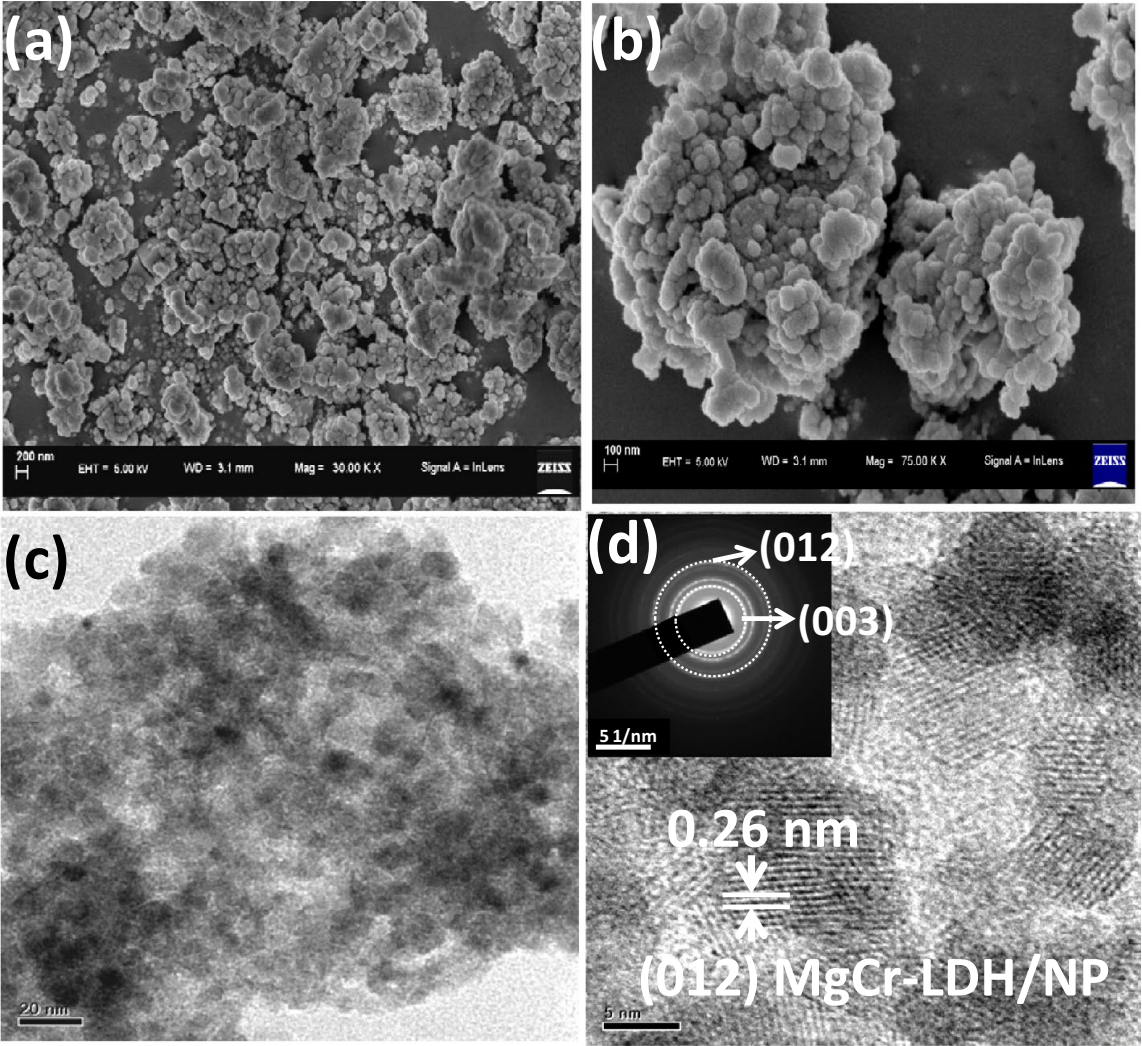
(**a**,**b**) FESEM morphology of MgCr-LDH/NP at different dimension, (**c**) TEM morphological features of MgCr-LDH/NP, and (**d**) lattice spacing of MgCr-LDH/NP as identified from HR-TEM image and corresponding SAED pattern (in-set image) of the material structure.

The field emission scanning electron microscopy (FESEM) techniques were used to reveal the morphologies of the as-synthesized MgCr-LDH/NP. Figure 2a,b indicates the FESEM morphological analysis of MgCr-LDH/NP, representing the creation of normal 3D aggregated particle-like architecture contented with abundant hexagonal NS. Specifically, with the addition of 20 mL of HCHO and mild hydrothermal treatment of 80 °C and visible light exposure of 30 min, hexagonal NS as visualized in Fig. 2a, self-stacked to shaped into cluster and further looks like 3D NP structure consisting of self-stacked 2D NS as MgCr-LDH/NP (Fig. 2b, average d = 50 nm). Apparently, HCHO controlled the LDH morphology, and coordinated the CHO– ligand to the metal cations, plus the regulation of pH by release of H^+^ and OH^−^ from NaOH. The Mg^2+^ ions precipitate rapidly (Ksp = 1 × 10^–12^) by adding the solution associated with OH^−^ and NO_3_^−^, forming Mg(OH)_2_ that offer the nucleation site for Cr^3+^ ions to precipitate (Ksp = 1.6 × 10^–30^) as Cr(OH)_3_.Though, Mg^2+^ and Cr^3+^ ions coordinated with CHO^−^ ions and generated [Mg (CHO)_x_]^2−x^ and [Cr(CHO)y]^3−y^ intermediates, the H^+^ and OH^−^ ions neutralize to fix the solution pH at 7. In these circumstances, nucleation and growth process of LDH by reacting with OH^−^ and NO_3_^−^ could be arrested, leading to the creation of MgCr-LDH/NS. The MgCr-LDH/NP would be generated by self-assembly of freshly created MgCr-LDH/NS (Fig. 2a) on the previously formed layers (Fig. 2b). As discussed, the layered 2D MgCr-LDH/NS interconnected to create 3D NS consisting of 2D NS with an open structure; besides, these kind of morphological aspects furnish an enormous amount of available surface sites, which manifest enrich photo/electroactive sites for the water redox reaction, and open space for ion pooling for escalating the kinetics of diffusion barrior within the electrode/electrolyte interface^78^.

**Figure 3:**
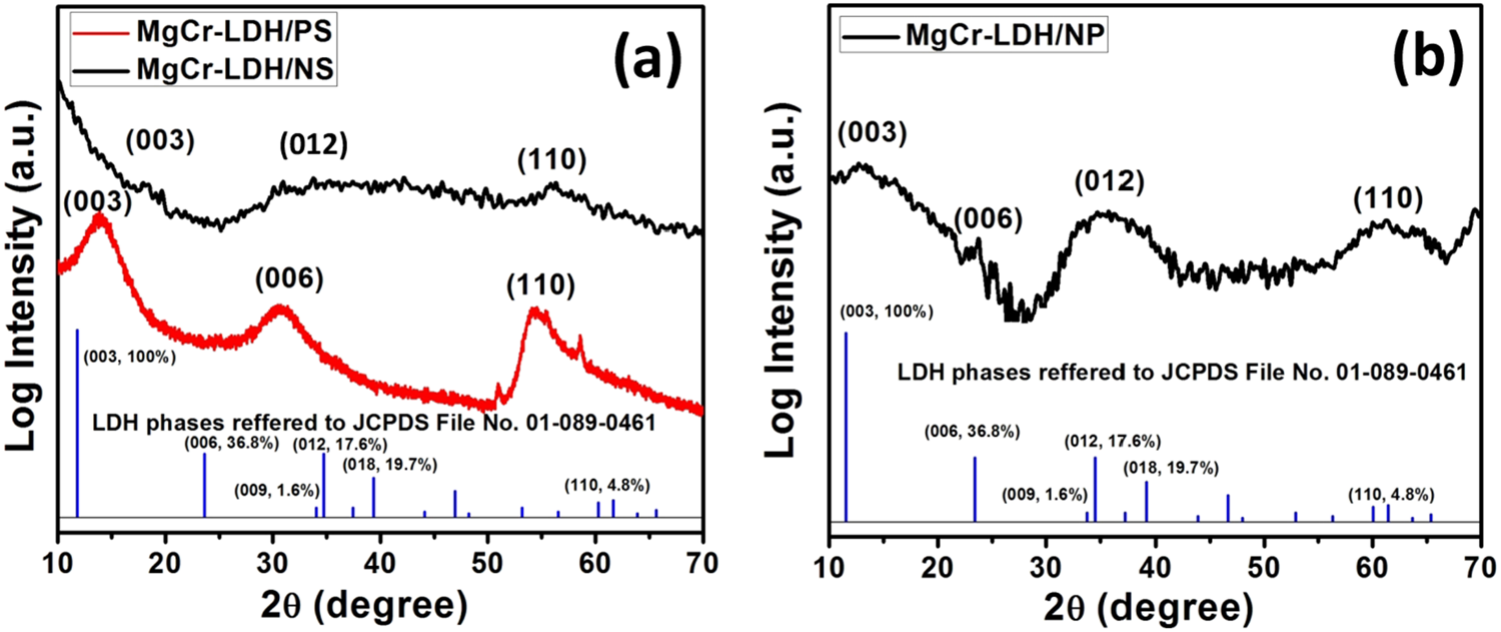
(**a**) PXRD spectra of MgCr-LDH/PS and exfoliated MgCr-LDH/NS; (**b**) PXRD spectra of MgCr-LDH/NP under the influence of hydrothermal treatment of 80 °C and visible light exposures of 30 min.

Following the FESEM analysis, the structural aspects of the 3D MgCr-LDH/NP, could be well-recognized vide transmission electron microscopy (TEM) and high resolution-TEM (HR-TEM) analyses. TEM images of MgCr-LDH/NS (Supplementary Fig. S1a,b), and MgCr-LDH/NP (Fig. 2c) elucidate the effect of HCHO induced mild hydrothermal treatment and visible light irradiation on structure and morphologies of materials. Figure 2c exemplified the distinct and fluffy nature of the characteristic 3D MgCr-LDH materials. Further the TEM image also illustrated the consistency of dense and thin 2D NS (Supplementary Fig. S1a,b), in typical 3D MgCr-LDH/NP (Fig. 2c)^79^. The free and exposed 2D NS surface ease out catalyst reactions and triggers the photocatalytic water splitting activities of binary MgCr-LDHs^80^. Furthermore, the obscure part appeared in Fig. 2c was owing to the dense stacking, and distortation of the NS and these properties could be identified in graphene and analogus materials^81^. The high resolution-transmission electron microscopy (HR-TEM) images of MgCr-LDH/NP (Fig. 2d) offers a distinct view of lattice distance ~ 0.26 nm, represented by dotted lines, which is approximately matching with the typical (012) plane in 2D MgCr-LDH NS. The particle diameter of MgCr-LDH/NP is assumed to be average distance of 20–50 nm. A similar morphological pattern is also detected in NiAl LDHs^54^ etc. The inset selected area electron diffraction (SAED) pattern (Fig. 2d) also confirms the (003), and (012) planes of the LDH fully matching with the X-ray diffraction (XRD) pattern (Fig. 3). These results signify the polycrystalline nature of the NS in the NP of the single system binary LDHs^54^. Furthermore, the sharp contrast elemental mapping of the Mg, Cr, and O together with the energy dispersive X-ray (EDX) spectral plot clearly specify the uniform allocation of constituent elements in MgCr-LDH/NP (Supplementary Fig. S2a–d).

### Structural, surface area and valence state features of MgCr-LDHs

The solid state crystallographic planes of MgCr-LDHs based samples were characterized through powder XRD (PXRD) pattern and the entire diffraction pattern could be resembled into hexagonal crystal phase with space group R3m of rhombohedral symmetry of hydrotalcite like materials (Fig. 3). The diffraction pattern of MgCr-LDH/PS (Fig. 3a), consisting of three main peaks at 2Ɵ = 13.9°, 30.8°, and 55.0° could be ascribed to the phase reflection of (003) plane, edge plane of (006), along with (110) plane^38,82^, which are approximately matching with the JCPDS file No. 01-089-0461. The (012) and (110) edge planes in XRD pattern of LDH are considered as the main exposed planes and match up to the cationic and anionic distances within the layered structure. The peak index of the (110) reflection plane was found approximately at 2Ɵ = 55.0° and evidence the retaining of the LDH layered structure^38^. The higher shifting of the (003) and (006) planes together with the lower shifting of (110) basal reflection plane suggested a change in the unit cell parameter and decline in the periodicity of basal planes. This is related to the H_2_O content from the interlayer LDH galleries. The interlayer-spacings (d) were calculated by the use of Braggs law, nλ = 2d sin (Ɵ), where n = 1, λ = wavelength of the target, and Ɵ = incidence angle. The d-spacing value of MgCr-LDH/PS related to (110) plane was calculated to be 1.66 Ȧ, which is of typical characteristic of NO_3_^−^ intercalated LDH materials.

Alternatively, the PXRD pattern of MgCr-LDH/NS (Fig. 3a), clearly disclosed broad and symmetrical basal reflections at lower 2Ɵ = 34.4°, corresponding to the (012) basal planes with little spike type of asymmetrical reflections at higher 2Ɵ = 56.9°, assigned to the (110) planes owing to the HCHO induced exfoliation process, which are partially matching with the JCPDS file No. 01-089-0461. The reduced intensity and significant higher shifting of the (003) plane at 2Ɵ = 18.3°, and missing of the intense (006) planes compared to MgCr-LDH/PS, indicated with the interlayer height differences, and change in basal spacings and stacking disorder due to the variation in water contents and formation of discrete nanosheets under the influence of HCHO induced exfoliation^76,77^. The d-spacing value of MgCr-LDH/NS related to the (110) plane was calculated to be 1.6169 Ȧ.

In contrast, the MgCr-LDH/NP (Fig. 3b), exhibits sharp and broad reflection of the main exposed planes of (003), (012) and (110) at 2Ɵ = 12.9°, 34.7° and 60.6°, respectively (JCPDS file No. 01-089-0461). The relatively shifting of the broad reflections peaks of MgCr-LDH/NP to higher 2Ɵ angle in comparison to MgCr-LDH/NS is quite indicative of the decrease in the interlayer distance, which is an indicative of the cross-assembling of the nanosheets and corresponding evolution of the bundles of nanoparticles in 3D open structure. This consequences are further verified by the decrease in interlayer distance of 1.5267 Ȧ relative to the (110) basal planes of MgCr-LDH/NP. Furthermore, the slight and less intense growth of the (006) basal reflection planes at 2Ɵ = 23° demonstrate a reduction in periodicity of basal reflection plane owing to the cross-association of nanosheets to form bundles of nanoparticles. This implies that the crystal sizes are reduced in both lattice parameter *a* (a = 2d(110)) and *c* (c = 3d(003)) directions, indicative of self-stacking thickness of LDH nanosheets in 3D assembly of nanoparticles. These results showed that there were no other impurity phases detected in the PXRD pattern of MgCr-LDH based nanostructure materials during the structural variation from MgCr-LDH/PS to MgCr-LDH/NP through MgCr-LDH/NS. The variations in crystallographic information of MgCr-LDH based samples are included in Table S1.

The Fourier transform infrared (FT-IR) spectroscopy (Supplementary Fig. S3) also exploits the alteration of molecular units during the formation of MgCr-LDH/NP. In the case of MgCr-LDH/NS, the strong and broad band located at exactly 3400 cm^−1^ corresponds to the superposition of –OH stretching mode of vibrations, underneath the subsistence of –OH functional group of metal hydroxide layers^16,21,83^. The extremely weak broad band located at approximately 3000 cm^−1^ is accredited to the existence of hydrogen bonds among H_2_O molecules residing in the interlayer and –OH group of metal hydroxide layers^84^. In addition; an additional absorption band at 1642 cm^−1^ corresponds to the deformation of H_2_O molecules^85^. The insignificant band at 652 cm^−1^ collectively with the band at 1450 cm^−1^ are related with the overlapped NO_3_^−^ bending mode of vibration with the unwanted carbonate groups perhaps contaminated from the CO_2_ gas of air^86^. Mostly absorption bands beneath 800 cm^−1^ can be accredited to M–O bending and stretching mode of vibrations. In the interim, the FT-IR spectra of MgCr-LDH/NP signifies extremely broad shoulder peak of –OH functional group at 3400 cm^−1^ and the missing peak of –OH group approximately at 3000 cm^−1^, demonstrating that the coordinated –OH groups with the metal cation of hydroxide layers exist in different phase and possess defect sites. Furthermore, the decrease of the peak intensity at 1450 cm^−1^ and 652 cm^−1^ signifies that NO_3_^−^ and CO_3_^2−^ anions are completely eliminated after a hydrothermal treatment and light irradiation^85^. Similarly, the absorption bands beneath 800 cm^−1^ in MgCr-LDH/NP can be accredited to the M–O bending and stretching mode of vibrations. As the LDH layered structure is stabilized by the electrostatic interactions among the hydroxide layer and intercalated anions; so the elimination of NO_3_^−^ anion in MgCr-LDH/NP by stumbling of formamide assisted hydrothermal and light treated exfoliation usher to diminish their interactions, which in succession causes delamination of cationic layers and further self-assembling of the nanosheets and aggregated into prosper like a nanoparticles in 3D structure as confirmed from the TEM analyses of the material structure (Fig. 2c).

**Figure 4:**
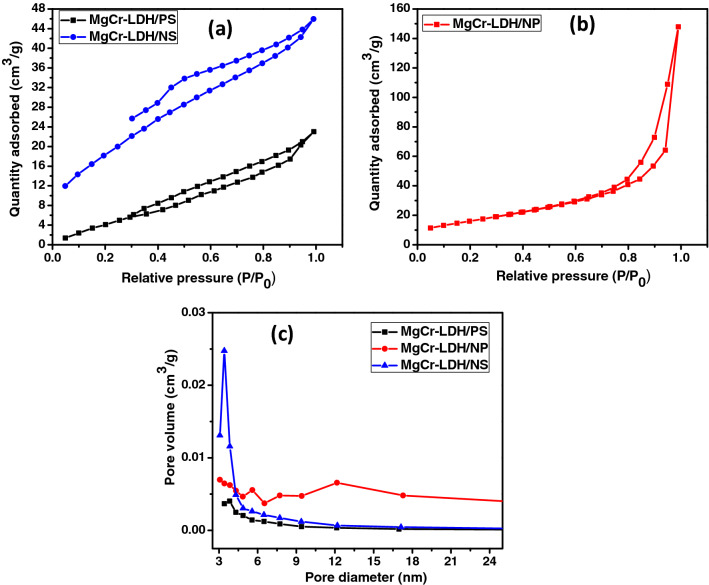
(**a**) N_2_ sorption isotherm; (**b**) pore size distribution of mesoporous MgCr-LDH/PS, MgCr-LDH/NS and MgCr-LDH/NP.

The as-synthesized MgCr-LDH based materials were further characterized by the N_2_ adsorption–desorption isotherms (relative pressure (P/Po) vs. volume of N_2_ adsorbed) in order to study the surface area, the average pore volume and the mesoporosity nature of the samples, which could have remarkable effect on the electrochemical properties and photocatalytic performance of the materials. All of these materials displayed type-IV isotherm with H1 hysteresis loop and shows mesoporous characteristics. The BET surface area of MgCr-LDH/PS, MgCr-LDH/NS and MgCr-LDH/NP were found to be 45, 96 and 115 m^2^/g respectively (Fig. 4a,b). The pore diameter of the as-synthesized MgCr-LDH/NP material are found to be 5.57, 7.71, 12.13 nm and supports the mesoporous nature as similar to its nanosheets and pristine materials as depicted in Fig. 4c. The increased volume of mesoporosity in 3D MgCr-LDH/NP represents the secondary pores, which arises due to the swelling behavior of OH^−^ groups induced by HCHO together with removal of gaseous ions during the constant hydrothermal treatment at 80 °C for 12 h and visible light exposure for 30 min for the advancement of porous structure. Table 1, outlined the surface area, pore diameter and pore volume of the MgCr-LDH based materials. Hence, the wide pore volume of MgCr-LDH/NP indicates the mesoporosity characteristic with high surface area that promoted more reactive sites available at the surface of catalyst which could enhances the rate of water splitting reactions.

**Table 1:** BET Surface area, pore diameter and pore volume of the MgCr-LDH/PS, MgCr-LDH/NS and MgCr-LDH/NP samples.

Samples	Surface area (m^2^/g)	Pore diameter (nm)	Pore volume (cm^3^/g)
MgCr-LDH/PS	45	3.8	0.01
MgCr-LDH/NS	96	3.4	0.04
MgCr-LDH/NP	115	5.57, 7.71, 12.13	0.22

**Figure 5:**
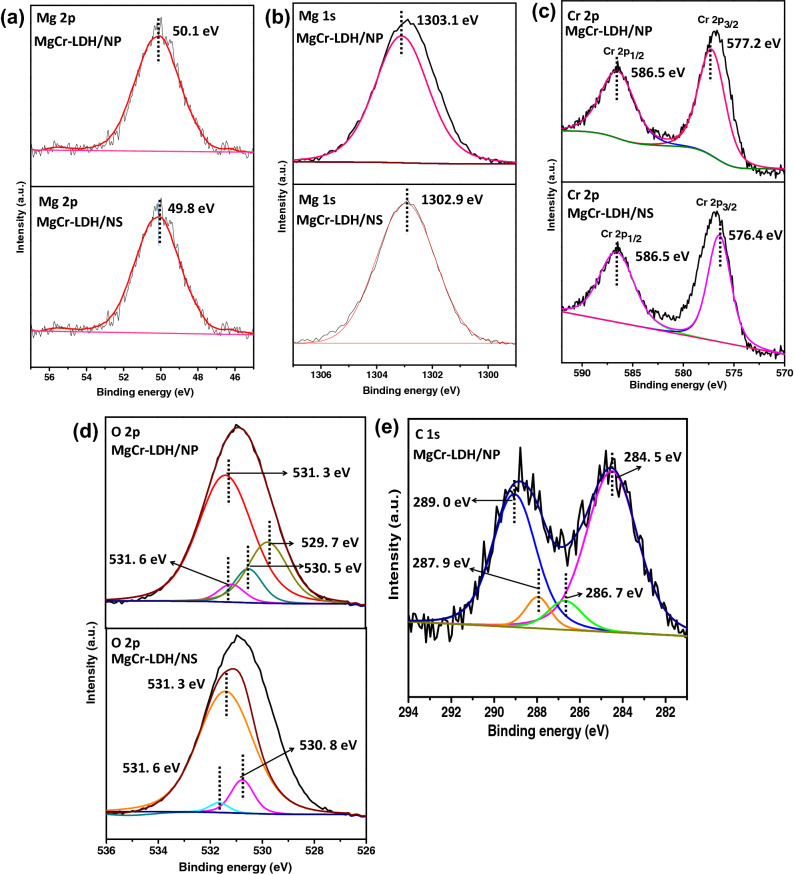
Analysis of the XPS results of the deconvoluted XPS spectra of MgCr-LDH/NS and MgCr-LDH/NP for Mg2p, Mg2s, Cr2p, O2p, and C1s.

The X-ray photoelectron spectra (XPS) elucidate the surface elemental composition and surface states of binary MgCr-LDH based catalysts (Fig. 5). The presence of Mg, Cr, O and C elements were noticeable on the XPS surface survey spectra (Supplementary Fig. S4). Figure 5 represents the deconvoluted Gaussian-fitted XPS spectra of Mg 2p, Mg 1s, Cr 2p, O 1s and C 1s in the modified MgCr-LDH/NS and MgCr-LDH/NP based materials. In an illustration, Fig. 4a shows the Mg 2p XPS spectra of MgCr-LDH/NS and MgCr-LDH/NP. In MgCr-LDH/NS, for the Mg 2p_3/2_ spectrum (Fig. 5a), peak located at 49.8 eV reveals the occupancy of Mg(OH)_2_ and corresponded to the main Mg^2+^ cationic states in the material^47^. Moreover, peak fitted Mg 2p_3/2_ spectrum of MgCr-LDH/NP shows the Mg^2+^-cationic states after the structural transformation into nanoparticles (Fig. 5a). However, the corresponding Mg 2p_3/2_ peaks of MgCr-LDH/NP were blue-shifted to higher binding energy 50.1 eV (difference in energy shifting ∼ 0.3 eV). The fitting XPS spectrum of Mg 2p in MgCr-LDH/NP reveals the existence of bivalent Mg^2+^ in material. Figure 5b, showed the appropriate binding energy of Mg 1s peak of MgCr-LDH/NS at 1302.9 eV^49^, whereas the Mg 1 s peak of MgCr-LDH/NP was identified at 1303.1 eV, which noted the absolute continuation of Mg^2+^ states in MgCr-LDH. Figure 5c showed the XPS spectrum of Cr 2p of MgCr-LDH/NS. The energy level fitted Cr 2p_3/2_ and Cr 2p_1/2_ peaks were appeared at 576.4 and 586.5 eV in the, respectively^49^. The binding energy of Cr 2p peak at 576.4 eV denoted the creation of Cr–O bond^70^. Similarly, the core-level Cr 2p XPS spectrum of MgCr-LDH/NP, could be fitted into two spin–orbit doublets, which corresponded to the peaks of Cr 2p_1/2_ and Cr 2p_3/2_ for the presence of Cr^3+^ cation^74^. The binding energy of Cr 2p at 577.2 and 586.5 eV was accredited to the Cr 2p_3/2_ and Cr 2p_1/2_ states, which verified the trivalent nature of Cr ions. The binding energy of Cr 2p at 577.2 eV proposed the generation of Cr–OH bond. These results suggested that the metal cations associated with MgCr-LDH/NP preserved the unusual valence state after the hydrothermal and light treatment. As illustrated in Fig. 5c, the Cr 2p peaks in MgCr-LDH/NP slightly shifted towards higher binding energy in comparison to the Cr 2p peaks in MgCr-LDH/NS. These results might be attributed to the successful introduction of Cr^3+^ with empty electron orbitals, which adjusts the electronic structure of the catalyst. Figure 5d displays the O 1s XPS spectra of MgCr-LDH/NS and MgCr-LDH/NP. The high resolution O 1s XPS spectrum of MgCr-LDH/NS could be deconvoluted into three peaks at 530.8, 531.3 and 531.6 eV, which are assigned for lattice oxygen linked with Mg and Cr metal, surface hydroxyl bonded to metal centers and oxygen vacancies or under-coordinated lattice oxygen vacancies^49^. Moreover, in comparison to the O1s spectrum of MgCr-LDH/NS, the approximate peaks identified in MgCr-LDH/NP includes 529.7, 530.5, 531.3, and 531.6 eV, which is associated for water molecules, lattice oxygen, surface –OH group, and oxygen vacancies, respectively^73^. Moreover, the more prominent oxygen vacancies peak in MgCr-LDH/NP signifies the subsistence of oxygen vacancies related to defects type owing to existence of delaminated MgCr-LDH during the hydrothermal process and further light irradiation causes aggregation of the nanosheets to produce MgCr-LDH/NP containing oxygen vacancies sites. Figure 5d, shows that the hydrothermal treatment enhances the intensity of the M–OH bond, and formation of oxygen vacancies on assembly of nanosheets in MgCr-LDH/NP becomes more favorable at an optimal light exposure time of 30 min. The percentages of Ov as determined from the fitted peak area of O 1s spectra are 25% and 50% for MgCr-LDH/NS, and MgCr-LDH/NP samples. Further hydrothermal treatment with light exposure causes appearance of new peaks attributed to the formation of adsorbed water peaks at 529.7 eV^73^. In addition, the positively shifted Cr 2p_3/2_ peak of MgCr-LDH/NP (~ 577.2 eV), demonstrated the decreases in electron density around Cr and electron clouds are inclined towards the Mg(OH)_2_ surface owing to the formation of oxygen vacancies. Additionally, the C 1s XPS spectrum of MgCr-LDH/NP (Fig. 5e) revealed the existence of C 1 s main peak with high binding energy at 289 eV corresponded to O−C=O linkage. The other binding energy peaks at 287.9, 286.7, and 284.5 eV corresponded to C–O–C, C–OH, C–C linkage, respectively^17^. All of these characteristic features substantiate that hydrothermal treatment of formamide treated bulk MgCr-LDH could led to the removal of gaseous products like NO_2_ from the interlayer of LDH and causes structural twist towards nanosheets with oxygen vacancies and further exposure under visible light resulted with self-aggregation and removal of other gaseous products like CO_2_, H_2_, and H_2_O in a sintered confinement, thereby leading to nanoparticles like MgCr-LDH/NP containing exfoliated self-stacked nanosheets with enriched oxygen vacancies. Hence togetherness of hydrothermal and visible light treatment has dramatic effect on structural twist from bulk MgCr-LDH to nanoparticles through nanosheets for significant PEC properties and photoinduced water splitting reactions.

### PEC properties studies of the MgCr-LDH material

**Figure 6:**
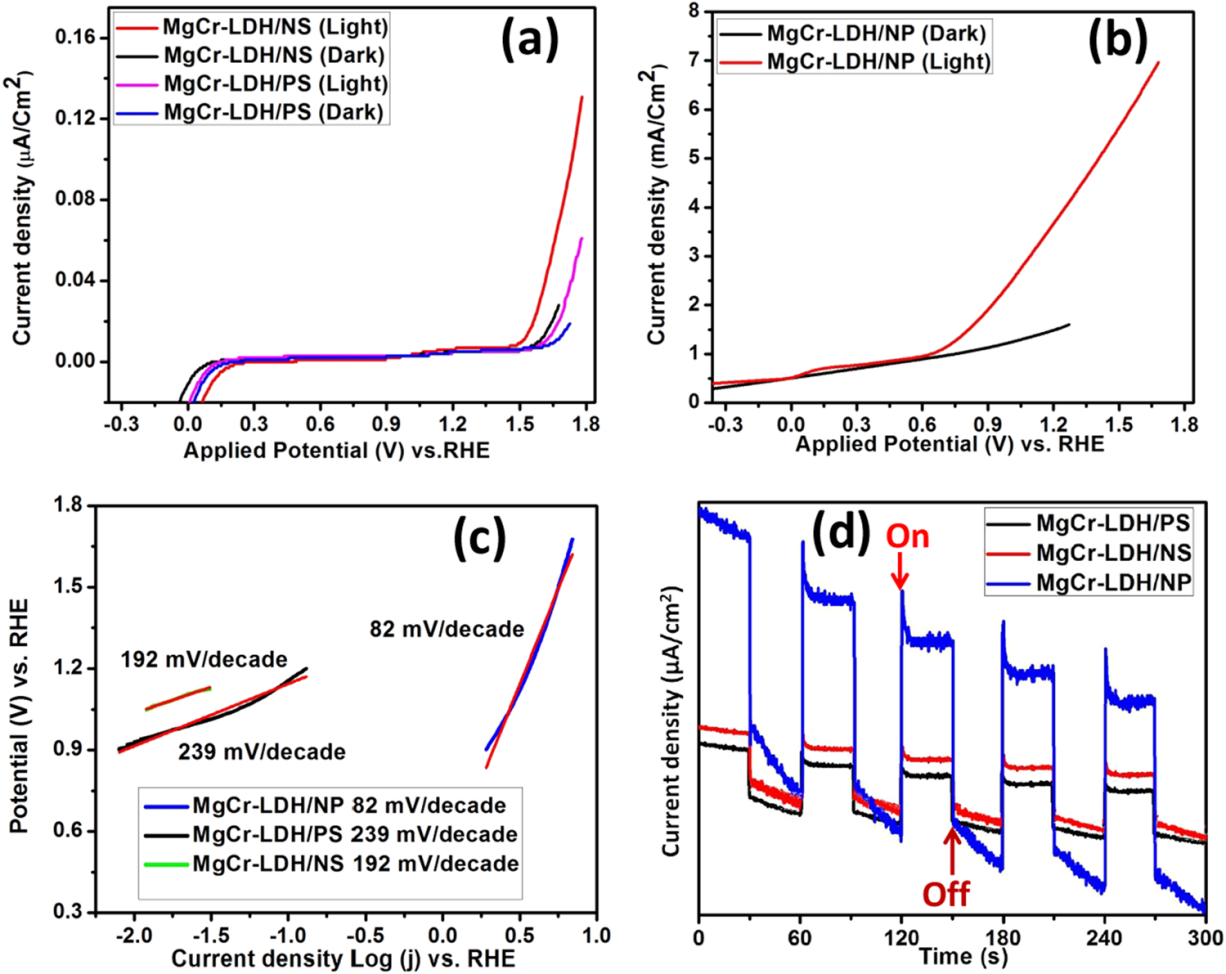
(**a**,**b**) Dark and light current density of MgCr-LDH/PS, MgCr-LDH/NS and MgCr-LDH/NP via LSV plot, (**c**) Tafel slope plot of MgCr-LDH/PS, MgCr-LDH/NS and MgCr-LDH/NP and (**d**) Transient photocurrent density of MgCr-LDH/PS, MgCr-LDH/NS and MgCr-LDH/NP measured through light on–off cycle.

The magnificent PEC photocurrent properties of the MgCr-LDH/PS and the corresponding MgCr-LDH nanosheets and hierarchical 3D MgCr-LDH/NP structure were investigated using LSV studies as obtained under dark and visible light illumination in order to legacy the function of photoinduced excitonic charge separation to intensify the net photocurrent as shown in Fig. 6a,b. The photocurrent measurement studies of the series of MgCr-LDH materials were recorded in potential panel of − 1.0 to 1.2 V vs. Ag/AgCl reference electrode using 0.1 M Na_2_SO_4_ (pH = 6.5), at scan rate of 10 mV s^−1^ and converted to reversible hydrogen electrode (RHE) according to the Nernst equation.

4 $$ E({\text{RHE}}) \, = E{\text{Ag}}/{\text{AgCl }} + \, 0.{197 } + \, 0.0{\text{59 pH}}. $$

Figure 6a,b, showed that MgCr-LDH/PS could able to produce the linear current density of 0.06 µA/cm^2^ at potential of 1.19 V vs. RHE under light exposure, which is much lower than MgCr-LDH/NS and MgCr-LDH/NP. In particular, the enhancement in photocurrent jumped to 6.9 mA/cm^2^ at 1.67 V for MgCr-LDH/NP with respect to 0.12 µA/cm^2^ at 1.77 V for MgCr-LDH/NS, and 0.06 µA/cm^2^ at 1.76 V for MgCr-LDH/PS electrode *vs.* RHE under scan rate of 10 mV/s. Meanwhile, the formation of nanosheets assembling in MgCr-LDH/NS at hydrothermal treatment of 80 °C and further assembling and aggregation of the nanosheets to nanoparticles like 3D structure under light treatment of 30 min, intensified the effective active sites for water oxidation, which suggested the synergistic effect of structural evolution of hierarchical nanostructure together with the beneficial role of the Cr^3+^ dopant and oxygen related defect sites for effective charge separation and incremental current density of the MgCr-LDH/NP^54^. At the meantime, no noticeable input from the dark current scan could detect for all electrodes in the entire investigated potential window. The onset potential is determined by the junction point of the light current density and dark current density in the j–V curve^87^. The generation of the photocurrent at the onset potential of a semiconductor photoelectrode explores their catalytic tendency towards redox activities. The shift in the onset potential of the nanostructured material reveals the structural transition with enhanced photoelectrochemical redox reaction activities. Particularly, low onset potential reveals the minimum loss of energy during the electrochemical redox reactions. The onset potentials in Fig. 5a are recorded at 0.9 V while Fig. 5b shows the onset potential at 0.2 V vs. RHE. The unusual big difference of onset potentials of approximately 700 mV was detected among these electrodes, which might be due to the three-dimensional structure of MgCr-LDH/NP containing dispersed nanoparticles, reducing the recombination of the charges and promotes the charge transfer. Hence, the onset potential of MgCr-LDH/NP is greatly decreased through the structural transition from bulk to nanosheets, and then to 3D morphological features of nanoparticles, which is indicative of the amalgamation of oxygen vacancies related defect sites for easy charge tunneling and fast separations of excitonic charge pairs for enhanced photocatalytic water oxidation performances.

The Tafel slope is mostly utilized to authenticate the superior OER properties of various binary LDH, which is considered as the rate determining step in the water splitting process; and is deliberate by below equation^88^.

5 $$ \eta \, = {\text{ a }} + {\text{ b log }}\left( {\text{j}} \right), $$

where ƞ, a, b, and j correspond to the overpotential, constant, Tafel slope, and current density. The Tafel slopes were rationalized from the LSV polarization curves at scan rate of 10 mV s^−1^ by plotting potential (V) vs. log (j) (RHE), where the LSV curves were counted at a particular region starting from the onset potential where current density starts to increasing. The linear fitting of the top portion of the Tafel plot gives rise to the Tafel slope. The calculated Tafel slopes are 239, 192, and 82 mV/decade for MgCr-LDH/PS, MgCr-LDH/NS and MgCr-LDH/NP, respectively (Fig. 6c). It was found that morphological variation from bulk to nanosheets and further nanoparticles like assembly decreases the Tafel slopes and the smallest slope was tenable for MgCr-LDH/NP, confirming the highest current density and faster kinetics towards water splitting reactions. Normally, lower overpotential and smaller Tafel slopes constituted better catalytic water splitting performance and well-recognition to the fast electron–hole transfer and separation process owing to extraneous and uncovered active sites in 3D MgCr-LDH/NP^73,88^. To further scrutinize the transient PEC response of MgCr-LDH-based photoelectrode, chopped light on and off irradiation with a cycle of 30 s at constant potential of 0.5 V was applied and recorded a polarization curves as shown in Fig. 6d, All the photoelectrode exhibit rapid transient responses in the illuminated on and off cycle, accompanying a faster photoinduced excitonic charge carrier generation route. It is recognized that photocurrent density quickly shows incremental in current density and then maintains it till the light source switched off and then suddenly falls to a steady value.

**Figure 7:**
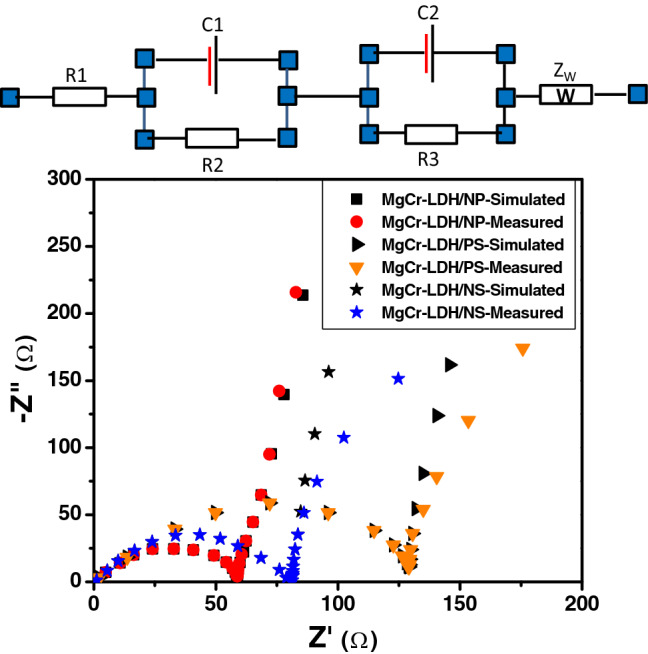
Electrochemical impedance spectra of the measured and simulated data of the as-synthesized MgCr-LDH/PS, MgCr-LDH/NS and MgCr-LDH/NP samples.

The electrochemical impedance spectroscopy (EIS) measurement studies were executed to investigate the charge-transfer resistance properties at the interface of photoelectrode and electrolyte for the advancement of the kinetics of electrode (solid)-electrolyte (liquid) reactions along with stability measurement of the MgCr-LDH based samples. The EIS results of MgCr-LDH based samples have been analyzed and the measured and simulated impedance data are described in the Nyquist plot as shown in Fig. 7. All EIS data’s were fitted with the Randles equivalent circuit as displayed in the upset image of Fig. 7. In the Nyquist plot of MgCr-LDH based electrodes, R1 is noted as the series resistance of the circuit, which is related to the charge-transfer resistance at the interface of Pt counter electrode/electrolyte at high frequency region of the semicircle. The R2 is noted as the charge-transfer resistance (Rct) at the interface of working electrode (MgCr-LDH)/electrolyte in the mid frequency region of the semicircle. The R3 is noted as the charge-carrier-transfer resistance in the Helmholtz double layer. The CP1, CP2 and Z_W_ correspond to the chemical capacitance and Warburg impedance, respectively. Normally, the electrochemical model circuit suggested that the minor semicircle portion is related to the charge transfer resistance (Rct) and the major straight line is relevant to the mass transfer resistance (Rm) at low frequency^89^. The as-obtained fitted values of Rct for MgCr-LDH/PS, MgCr-LDH/NS, and MgCr-LDH/NP photoelectrodes were found to be 129.18, 80.80 and 59.17 Ω cm^−2^, respectively. The as-obtained MgCr-LDH/NP displays smallest Rct value among the three types of MgCr-LDH-based photoelectrode, which indicate the efficient dynamics of carrier charge separation and rapid surface redox kinetics, occurred on the MgCr-LDH/NP photoelectrode and electrolyte interface. Moreover, the stability of the photoelectrode is highly necessary to secure high PEC efficiency of the materials. The stability of the MgCr-LDH/NP photoanode samples was tested performing chronoamperometric *J*-*T* curve measurements by applying a constant potential of 0.5 V to overcome the Ohmic losses in the electrolyte and metal contacts under visible light exposure for 6000 s (Supplementary Fig. S5). Interestingly, rational photocurrent stability preservation over a suitable period was exemplified for the MgCr-LDH/NP nanostructure photoelectrode.

**Figure 8:**
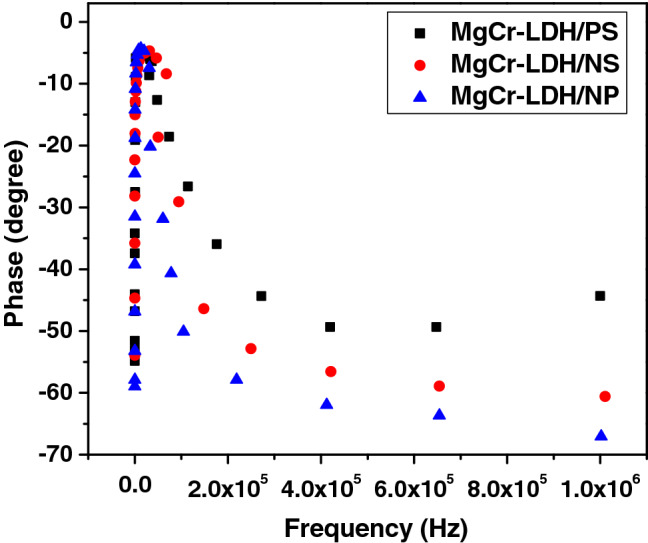
Bode Phase Plot of MgCr-LDH/PS, MgCr-LDH/NS and MgCr-LDH/NP.

On the other hand, the frequency dependent Bode phase angle plot of MgCr-LDH electrodes are shown in Fig. 8, and they are used to measure the electron lifetime in the nanostructured materials. Normally, the highest peak intensity of the Bode phase angle curve stipulates the rate of the charge transfer at the electrode interface. Figure 8 shows the highest peak maxima of the phase angle at low frequency region for MgCr-LDH/NP. The lifetime of photoinduced electrons at the electrode interface can be estimated using the relation *τ*_*n*_ = 1/(2*πf*_*max*_) where *f *_*max*_ is the frequency maxima. The MgCr-LDH/NP also reveals a peak shifting from high frequency region to low frequency region indicating the fast electronic transfer as *f*_*max*_ is directly proportional to the electron lifetime. The calculated τ values for MgCr-LDH/PS, MgCr-LDH/NS and MgCr-LDH/NP electrodes are 4.82, 9.98 and 12.75 µs, respectively. The large τ value of MgCr-LDH/NP electrode corresponds to the enhanced charge separation efficiency or minimum carrier recombination, owing to their morphological variation triggering superior PEC properties and improved photocatalytic water splitting performances.

**Figure 9:**
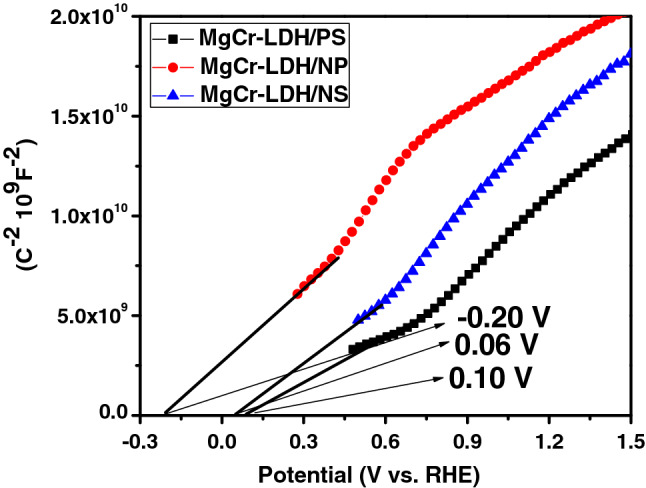
Mott-Schottky plot of MgCr-LDH/PS, MgCr-LDH/NS and MgCr-LDH/NP samples.

The Mott–Schottky plots were acquired for MgCr-LDH/PS and nanostructure MgCr-LDH photoelectrode samples indicating the reversed sigmoid plots resembling to n-type semiconductors (Fig. 9). A flat band potential (V_fb_) of an electrode could be calculated by following Mott–Schottky equation^47^,

6 $$\frac{1}{{c}^{2}}=\left(\frac{2}{q{\varepsilon }_{0}\varepsilon {N}_{d}}\right)\left({V}_{app}-{V}_{fb}-\frac{kT}{q}\right),$$

where *ε* is the dielectric constant*, N* is the the charge carrier density*, C* is the space charge layers capacitance, *V*a is the applied potential*, e* is the electron charge, and *ε*_0_ is the permittivity of vacuum. The estimated V_fb_ value recedes in the potential edge of CB (E_CB_) of n-type semiconductors *vs*. RHE. Furthermore, the carrier charges density (Nd) found from the Mott-Schottky plots was used to estimate the alteration in carrier charge concentration. The theoretical equation calculating Nd of semiconductor is as follows.

7 $$ {\text{Nd }} = \, \left( {{2}/\varepsilon \varepsilon^{0} .{\text{e}}} \right)\left[ {{\text{d}}\left( {{1}/{\text{C}}^{{2}} } \right)/{\text{dV}}} \right]^{{ - {1}}} . $$

Importantly, the flat band potential of MgCr-LDH/NP indicated decrease in band bending and higher slope assigned to the increased in carrier density, which is attributed to the defect-sites allowed to the charge transfer process among the electrode and electrolyte. Hence, the significant charge transfer rate in MgCr-LDH/NP photoelectrode is a synergistic result of 3D flower like structure containing 2D nanosheets and oxygen related defect sites. The cathodic shift of the conduction band edge of the MgCr-LDH/NP offers significant potential for water redox reaction under visible light exposure.

### Photocatalytic water splitting activity

**Figure 10:**
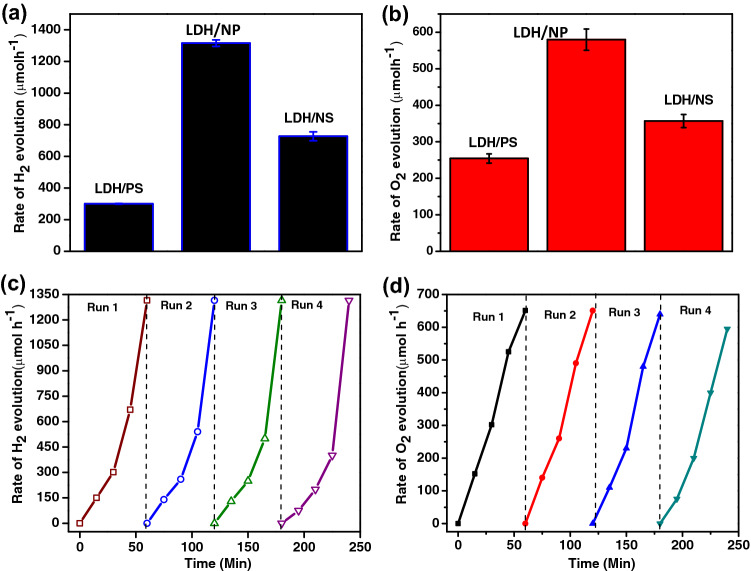
(**a**,**b**) Volume of H_2_ and O_2_ production plot under visible light illumination for 1 h; (**c**,**d**) Recyclability studies of MgCr-LDH/NP towards H_2_ and O_2_ evolution under visible light exposure.

The capability of the MgCr-LDH based samples to decompose water under visible light, have been tested for series of H_2_ and O_2_ production performance using home-made quartz cell test reactor closed with 125 W medium pressure Hg lamps emitting visible light with 1 M NaNO_2_ as UV cut off filter to expose light of λ ≥ 400 nm and Julabo based chiller under similar experimental conditions^16,17,21^. A power density of 100 mW cm^−2^ was precise for the visible light approach on the quartz chamber and the average light flux was ~ 2890 Lx. The dark experiment reveals no substantial H_2_ or O_2_ production either in the lack of catalyst or light, which notify that photocatalytic water splitting reaction is dependent on both catalyst and light. Firstly, the 0.03 g of MgCr-LDH/PS catalysts, MgCr-LDH/NS and MgCr-LDH/NP catalysts were tested for H_2_ and O_2_ evolution using 30 mL of 10 vol% aqueous solutions of CH_3_OH and AgNO_3_ as sacrificial agents. Figure 10a, reveals that as the structural transformation increases from the bulk phase to nanosheets and gradually increases towards nanoparticles, the H_2_ and O_2_ production shows a volcanic trend. The enhanced water splitting activity of binary MgCr-LDH/NP might be owing to the distinctive structural features (3D nanoparticles contented with self-stacked 2D nanosheets) and the synergistic effects among the dispersion of Mg, and Cr atoms as found from the TEM results. Figure 10a, shows the maximum hydrogen production of MgCr-LDH/NP reaches to 1315 μmol/h, which was 1.8 and 4.3 times of MgCr-LDH/NS (726 μmol/h) and MgCr-LDH/PS (300 μmol/h) under visible-light irradiation. This might be the results of the increase of electronegativity of MgCr-LDH/NP owing to the generation of oxygen vacancies. The exfoliation of MgCr-LDH under mild hydrothermal condition generates uncoordinated metallocentres and dense amount of free atoms at the edge sharing hexagon, responsible for oxygen related vacancies and causes intersection of the nanosheets for enhancing light harvestation ability of the materials and corresponding exciton separation efficiency directly or indirectly responsible for the water splitting reactions^54^. This is also reflected in the XPS spectra and impedance plots of the magnificent PEC properties, and the formation of nanoparticles structure is more conducive to H_2_ production because of the special structure of the layered 2D nanosheets inside the 3D nanoparticles offers added active phases, which amplify the excitonic separation process, so facilitates quick redox reaction. The existence of inconsistent oxidation states in the binary LDH (Mg2þ/1s and Cr2þ), due to the inclusion of Cr^3+^ in the framework, charge transfer, conductivity and electron capture hastily followed to facilitate the H_2_ production. Furthermore, the fabricated photocatalysts were also examined towards O_2_ evolution reaction (E^0^ O_2_/H_2_O =  + 1.23 V vs. RHE), under 250 W visible light emitting Hg-lamp for a period of 1 h^90^. Figure 10b showed that the MgCr-LDH/NP displayed the highest O_2_ production activity of 579 μmol/h followed by the MgCr-LDH/NS of 356 μmol/h and MgCr-LDH/PS of 254 μmol/h. The enhanced production capacity of MgCr-LDH/NP was due to the similar reason as explained for H_2_ production, i.e., owing to the presence of rich defect site related to oxygen vacancies trap out more photoexcited electrons, that would be available over CB of LDH matrix, separating out the holes at the VB of LDH under visible light illumination, which are then effectively channelized by the composed of 2D nanosheets in the hierarchal 3D structure of MgCr-LDH/NP^91^. Moreover, the 3D structures with high surface area intimately allocate the 2D active nanosheets, which could render additional active sites, and assist excitonic charge transportation due to porosity by the release of gaseous products in the 3D nanoparticles architecture. The addition of Cr^3+^ cations is supposed to be potentially redox active sites in the MgCr-LDH OER catalyst.

In order to measure the photostability of MgCr-LDH/NP catalyst during the H_2_ and O_2_ evolution, a cyclic H_2_ and O_2_ evolution experiment was carried out using 10% CH_3_OH and AgNO_3_ aqueous solution (Fig. 10c,d). Each cycle was 125 min, and total of 4th cycles was performed during the experiment. In the 3rd and 4th cycle, the H_2_ and O_2_ production gradually decreases due to the consumption of sacrificial reagents. The H_2_ and O_2_ evolution showed that the MgCr-LDH/NP photocatalyst possess good catalytic stability. In addition, XRD patterns were executed on the MgCr-LDH/NP photocatalysts before and after the cycle of H_2_ production, as shown in Supplementary Fig. S6. It was found that the XRD patterns of the catalyst before and after the H_2_ production cycle did not change significantly, except a little reduction in peak intensity which may be due to loss in catalyst handling, surface blocking by the sacrificial reagents and may be corrosion of catalysts surface during the catalytic reaction. Similarly, the TEM image of the MgCr-LDH/NP after fourth runs of the H_2_ evolution test reveals no significant changes in the phase and morphology (Supplementary Fig. S7). These features indicated that the MgCr-LDH/NP catalyst possess excellent water splitting activity.

**Figure 11:**
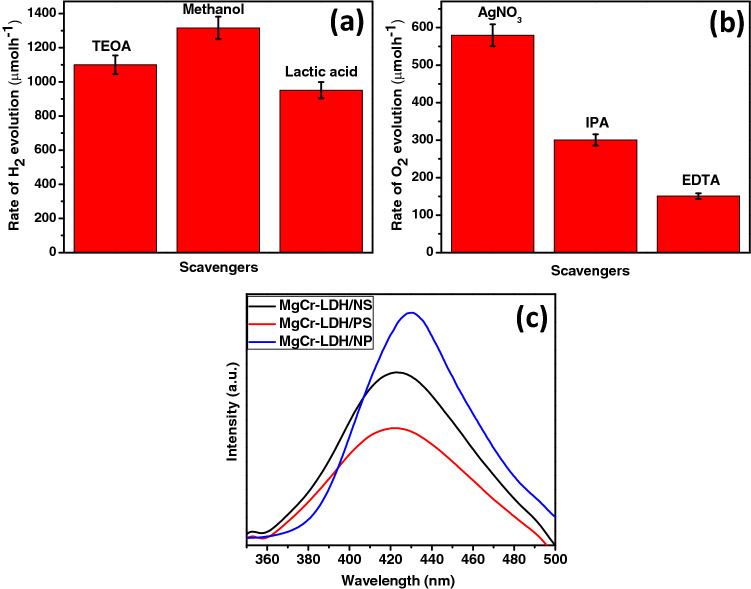
(**a**,**b**) Effect of different sacrificial agent on H_2_ and O_2_ evolution under visible light exposure, and (**c**) Hydroxyl radical test plot for all samples.

Additionally, the H_2_ production experiment of MgCr-LDH/NP was carried out under the presence of different sacrificial agent [10% lactic acid solution, 10% methanol, 10% triethanol amine (TEOA)] under similar experimental condition as shown in Fig. 11a. The sacrificial based water splitting reaction depends upon various factors such as the oxidation potential of the reagent, polarity, chain length, side-product formation, adsorption on catalyst surface, number of hydroxyl groups etc. Experiments showed that the highest H_2_ production was with the 10% CH_3_OH aqueous solution. This is because of the easy electron donor in the reaction system, and more electrons are generated and transferred to the active part of the photocatalyst for H_2_ generation reaction; further the reagent oxidized by photogenerated holes in the VB of LDHs to CO_2_. The mechanism detailed is as predicted in the following equations.

8 $$ {\text{MgCr}} - {\text{LDH }}\left( {{\text{CrO}}_{{6}} } \right) \, \to {\text{ h}}\nu ,{\text{ Catalyst }} \to {\text{ h}}^{ + } + {\text{ e}}^{ - } , $$

9 $$ {\text{2H}}_{{2}} {\text{O }} + {\text{ h}}^{ + } \to { 2} \cdot {\text{OH }} + {\text{ 2H}}^{ + } , $$

10 $$ {\text{e}}^{ - }_{{{\text{CB}}}} + {\text{ 2H}}^{ + } \to {\text{ H}}_{{2}} , $$

11 $$ {\text{CH}}_{{3}} {\text{OH }} + \cdot {\text{OH }} \to \cdot {\text{CH}}_{{2}} {\text{OH }} + {\text{ H}}_{{2}} {\text{O,}} $$

12 $$ \cdot {\text{CH}}_{{2}} {\text{OH }} \to {\text{ HCHO }} + {\text{ H}}^{ + } + {\text{ e}}^{ - } , $$

13 $$ {\text{2H}} + \, + {\text{ 2e}}^{ - } \to {\text{ H}}_{{2}} , $$

14 $$ {\text{HCHO }} + {\text{ H}}_{{2}} {\text{O }} \to {\text{ HCOOH }} + {\text{ H}}_{{2}} , $$

15 $$ {\text{HCOOH }} \to {\text{ CO}}_{{2}} + {\text{ H}}_{{2}} , $$

16 $$ {\text{CO}}_{{2}} +_{{}} {\text{H}}_{{2}} {\text{O}}_{{}} \to {\text{ CH}}_{{4}} . $$

Further, scavenger experiment was performed to trace out the active species responsible for water oxidation by using different sacrificial agents such as AgNO_3_, isopropyl alcohol (IPA), ethylenediamine tetraacetic acid (EDTA-2Na) as displayed in Fig. 11b. It was pragmatic that the O_2_ formation activity is maximum in case of AgNO_3_, whereas on addition of IPA, and EDTA-2Na (hole scavenger), the reduction performance increases which indicates the active role of hole in the water oxidation process. Yet again, for quantifying the efficiency of the photocatalyst towards O_2_ production, the apparent conversion efficiency, was measured to be for photocatalytic O_2_ evolution by MgCr-LDH/NP system under visible light irradiation. Considering this results, the ·OH radical formation was experimented over different as-synthesized samples (MgCr-LDH/PS, MgCr-LDH/NS, and MgCr-LDH/NP) and the result depicted the highest possible formation of ·OH radicals, signifying the most resolute photoluminescence (PL) peak of the terephthalic acid (TA)-OH complex over MgCr-LDH/NP as shown in Fig. 11c. The ·OH formation ability of the MgCr-LDH/NP could be regarded as the effective separation of excitonic pairs via appropriate amount of oxygen vacancies and Cr^3+^ dopant for enhancing the kinetics of water oxidation leading to greater accumulation of highly oxidizable holes in the VB of the concerned material. Moreover, the calculated VB potential of MgCr-LDH/NP was approximately 2.0 eV *vs*. NHE, which is quite sufficient enough to generate ·OH radical (OH/·OH = 1.99 eV vs. NHE). Hence the formation of e^−^, h+ and ·OH radical is quite feasible over the surface of MgCr-LDH/NP for superior photocatalytic water splitting performances.

17 $$ {\text{MgCr}} - {\text{LDH}}/{\text{NP }} + {\text{ h}}\nu \, \to {\text{ MgCr}} - {\text{LDH}}/{\text{NP}}* \, \left( {{\text{h}}^{ + } + {\text{ e}}^{ - } } \right), $$

18 $$ {\text{4h}}^{ + }_{{{\text{CB}}}} + {\text{ 2H}}_{{2}} {\text{O }} \to {\text{ O}}_{{2}} + {\text{ 4h}}^{ + } , $$

19 $$ {\text{4e}}^{ - } + {\text{ 4Ag}}^{ + } \to {\text{ 4Ag}}^{0} , $$

20 $$ \left( {{\text{Ag}}^{0} } \right)_{{\text{n}}} \to \, \left( {{\text{Ag}}} \right)_{{\text{n}}} , $$

21 $$ {\text{4h}}^{ + } + {\text{ Ag}}^{ + } \to {\text{ Ag}}^{{{2} + }} , $$

22 $$ {\text{2Ag}}^{{{2} + }} + {\text{ 2H}}_{{2}} {\text{O }} \to {\text{ Ag}}_{{2}} {\text{O}}_{{2}} + {\text{ 4h}}^{ + } , $$

23 $$ {\text{Ag}}_{{2}} {\text{O}}_{{2}} + {\text{ 2h}}^{ + } \to {\text{ 2Ag}}^{ + } + {\text{ O}}_{{2}} . $$

### Insight into the possible photocatalytic mechanism of charges separation

**Figure 12:**
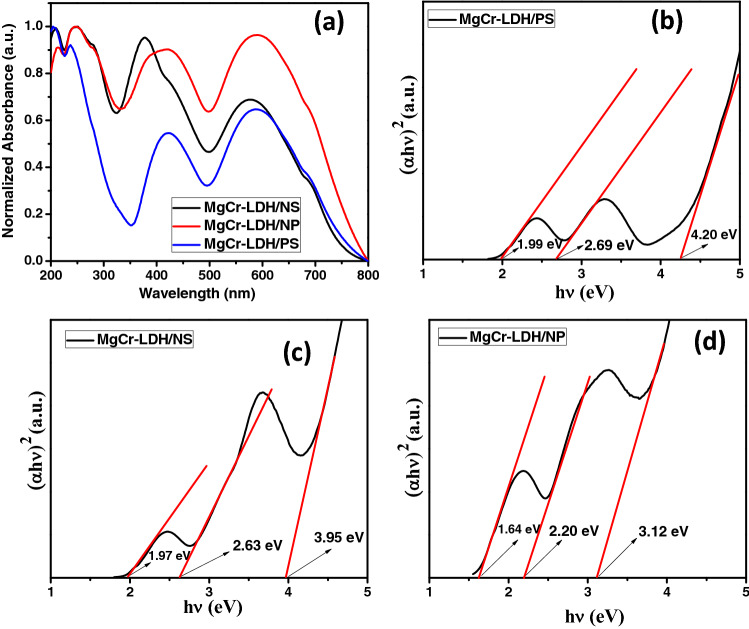
(**a**) Normalized UV–Vis DRS spectra of MgCr-LDH/PS, MgCr-LDH/NS, and MgCr-LDH/NP. (**b**–**d**) Band gap energy plot of MgCr-LDH/PS, MgCr-LDH/NS, and MgCr-LDH/NP as derived from Kubelka–Munk equation through Tauc plot.

Ultraviolet (UV)–visible (Vis) diffuse reflectance spectra (DRS) and PL spectra were analyzed to explore the optical properties and electronic charge transfer path within the MgCr-LDH based photocatalyst^16,17,21^. The optical absorption properties of a photocatalyst/photoanode are an important phenomenon, which directly affect their photocatalytic performances^92^. The UV–Vis absorption spectra of the MgCr-LDH/PS, MgCr-LDH/NS, and MgCr-LDH/NP photocatalyst are shown in Fig. 12a. All MgCr-LDH based samples exhibits strong optical absorption band in the wavelength range of 200–350, 400–500 and 550–700 nm, thereby possess the properties to perform as photocatalysts under visible light exposure. Most-importantly, MgCr-LDH/PS displays strong absorption band at 211–235 nm in the UV zone, which might be attributed to the ligand to metal charge transfer (LMCT) within the matrix of MgO_6_ and CrO_6_ octahedron in MgCr-LDH lattice i.e. O-2p → Mg-2p/Mg-1s orbital and O-2p → Cr-3dt_2g_ transition in octahedron surroundings^49^. Then absorption band in the spectral region at approximately 470–700 nm arises due to the charge transfer related to d-d transition of Cr^3+^ i.e. 3dt_2g_ → 3 deg orbital of which occupied with unfilled high energy 3 deg and under the exposure of visible light, the electronic transfer triggers from partial filled 3dt_2g_ orbitals to the 3 deg orbitals, sequentially^51^. Interestingly, the UV–Vis absorption spectra of MgCr-LDH/PS bulge from 450 to 750 nm, which could be accredited to the metallic-green color of MgCr-LDH. Alternatively, the UV–Vis-DRS spectrum of MgCr-LDH/NS catalysts, reveals a more intense hump at 378 nm, and 578 nm, verified with the excitonic transition band of O-2p → Mg-2p/Mg-1s orbital, O-2p → Cr-3dt_2g_ and d–d transition band in the excitonic states of 4A_2g_ → 4T_2g_ (F), respectively. The red shifted and intense absorption edge of MgCr-LDH/NS in comparison to MgCr-LDH/PS resulted owing to the reduced thickness of the exposed atomic sites of the nanolayers that minimized the electronic transfer distance and formation of oxygen vacancies as revealed from the XPS spectra, which allowed for dense concentration of electronic clouds over the nanosheets with enhanced conductivity for photoinduced catalytic performances.

Intriguingly, MgCr-LDH/NP, owing to the dynamics in structure with more defect sites endorsed numerous lights to scattered inside the folded and aggregated nanosheets to strengthen the optical path. The most interesting findings of MgCr-LDH/NP are of red-shifted light absorption intensity in comparison to MgCr-LDH/NS and MgCr-LDH/PS, respectively. Moreover, the intense defect site in terms of oxygen vacancies in MgCr-LDH/NP, amplify the absorption of light intensity in the wider visible zone for enhanced photocatalytic performances. As displayed in Fig. 12a, the optical absorption shoulder of MgCr-LDH/NP was situated in the UV–Vis region and displayed three types of absorption band i.e. inherent LMCT band among O-2p → Mg-2p/Mg-1s and O-2p → Cr-3dt_2g_ within 200–390 nm, d–d transitions of Cr^3+^ in the band region of 400‒712 nm. The d–d transitions of Cr^3+^ ion (d^3^ electronic arrangement), comprises of 4A_2g_ → 4T_1g_(F) and 4 A_2g_ → 4T_2g_(F) which were associated to the absorption peaks at 410 and 570 nm, respectively^45,93,94^. The predominant band of CrO_6_ absorption peak in the MgCr-LDH/NP arises owing to the atomic level variation among MgO_6_ and CrO_6_ with defects riched self-assembled and aggregated nanosheets. This intrinsic absorption band of MgCr-LDH/NP indicates the existence of lately twisted energy levels owing to oxygen vacancy among the conduction band (CB) and valence band (VB) of the material. The missing of surface oxygen of MgCr-LDH/NP encourage the easy charge pair transfer and separation as verified from the improved PEC properties, which results in enhanced photocatalytic water splitting performances of materials.

The band gap value of MgCr-LDH/PS, MgCr-LDH/NS, and MgCr-LDH/NP was obtained by using the Kubelka–Munk equation:

$$ \alpha {\text{h}}\nu \, = \,{\text{A }}\left( {{\text{h}}\nu \, - {\text{E}}_{{\text{g}}} } \right)^{{{1}/{2}}} , $$

where α is the absorption coefficient, hν is the incident photon energy, A is a constant, E_g_ is the band gap energy, respectively. The plot of (αhν)^2^ as Kubelka–Munk function *vs.* hv as function of photon energy gives the band gap energy value of MgCr-LDH based samples by using linear plot ranges extrapolated to the hv axis intercept (Fig. 12b–d). LDHs appeared to have a multifaceted band structure, which could be ascribed to the multiple band gaps, notifying the occurrence of different types of electronic transitions within the material^16,17,21,45^. Similar structure was identified in MgCr-LDH, howbeit it displayed three optical bandgap related to three absorption bands and accounts for directly allowed transition as versified from Fig. 12a–d. The three band gap values of MgCr-LDH/PS, MgCr-LDH/NS and MgCr-LDH/NP were calculated as (1.99 eV (*E*_*g*_*1*), 2.69 eV (*E*_*g*_*2*), 4.20 eV (*E*_*g*_*3*)), (1.97 eV (*E*_*g*_*1*), 2.63 (*E*_*g*_*2*), 3.95 eV (*E*_*g*_*3*)), and (1.64 eV (*E*_*g*_*1*), 2.20 (*E*_*g*_*2*), 3.12 eV (*E*_*g*_*3*)), respectively. Mostly, the *E*_*g*_*1* and *E*_*g*_*2* could be allocating to the d–d transitions of Cr^3+^ ion and the *E*_*g*_*3* could be set for the electronic transition from O*2p* → Mg*ns*/*np* and O*2p* → Cr*nd* levels. The lowest energy peak (E_g_1 = 1.64 eV) could be considered for the MgCr-LDH/NP photocatalyst as such a narrow band gap energy triggers for the maximum light harvestation ability of the material. However, based on the catalytic performance results of the material, we anticipated that the medium band gap energy of 2.20 eV and the related band alignment (CB and VB position) could be best suited for the MgCr-LDH/NP to drive the water splitting reactions as narrow band gap energy might causes fast recombination of electron–hole pairs under the exposure of visible light.

In addition, the band gap alteration of the as synthesized MgCr-LDH/NP is influenced by the defect site specific to oxygen vacancies, which could enhances the light absorption intensity in the visible region for significant photocatalytic water splitting performances.

**Figure 13:**
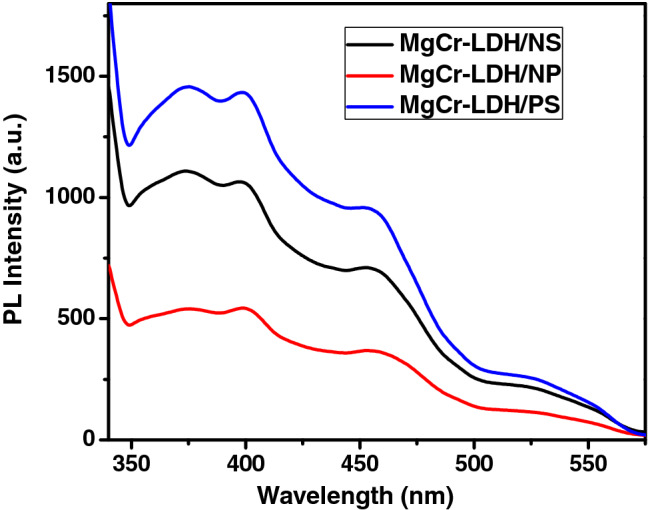
PL spectral plot of MgCr-LDH/PS, MgCr-LDH/NS and MgCr-LDH/NP.

Photoluminescence (PL) spectral technique is a fundamental tool to analyze the transfer and separation efficacy of photoinduced excitonic charge pairs in various semiconductor photocatalytic materials^16,17,21,45^. When the molecule absorbs light energy, first it would become in the excited state. However, the electrons in excited state have a short lifespan. If they do not react in time, they would be dissipated in the form of fluorescence and heat and the utilization rate of visible light of the catalyst might be reduced. The faster is the quenching of molecules in excited state of electrons, then higher the steady-state fluorescence emission peak intensity of the molecule (Fig. 13). The weaker PL signal signifies the higher lifetime of photogenerated charge carriers in semiconductor photocatalyst. Herein, PL was used to investigate charge transfer behavior of structurally evolved MgCr-LDH based materials starting from the bulk phase to nanosheets and then nanoparticles at an excitation wavelength of 320 nm as shown in Fig. 13^45^. The main peak of MgCr-LDH/PS is centered at approximately λ = 374 to 410 nm, which is associated with the typical photoemission of MgCr-LDH, approximately close to the bandgap energy of 3.7 eV (*E*_*g*_*1*)^49^. The emission peak at 400–410 nm in MgCr-LDH/PS is due to the vacancies in MgO_6_ octahedron, which acts as recombination sites and used to trap holes. The emission peaks at 459 nm could be linked to the radiative recombination of surface trapped localized excitonic charge carriers. The large decrease in PL intensity for MgCr-LDH/NP indicated that the recombination of photogenerated exciton pairs is significantly quenched owing to the large density of formation of defect sites and oxygen vacancies after the structural evolution from bulk to nanosheets and then self-assembling of the nanosheets led to the formation of nanoparticles^91,94^. This is related to the dynamic of charge transfer within the MgCr-LDH/NP matrix, which could be helpful to stimulate the PEC properties and corresponding water splitting reaction. The PL spectra of MgCr-LDH/NS and MgCr-LDH/NP also reveals three types of characteristic emission band comprising of vacancies exist in MgO_6_ octahedron of the Mg(OH)_2_ layers, localized surface defect, and oxygen vacancy. The localized defect state and oxygen vacancies in MgCr-LDH/NS arises owing to the presence of uncoordinated metal centers during the formation of nanosheets and triggers charge transfer inside the Mg(O)_6_ octahedron and towards the Cr(O)_6_ octahedron. However, the rich defects sites and oxygen vacancies peaks of MgCr-LDH/NP was identified at 500 nm and 524 nm, respectively, which was due to the occupancy of the numerous folded nanosheets during the secondary growth period of nanoparticles structure to reduce their surface energy, and release of the strong stress, under exterior forces for instance electrostatic, van der Waals forces, and hydrogen bonds in which twisted nanosheets self-assembled into stable and irregular 3D nanostructures^91^. MgCr-LDH/PS displays the strongest PL peak signifying higher efficiency of excitonic recombination process. The most diminished PL peak of MgCr-LDH/NP at about 373–500 nm reveals the lower recombination rate of photoinduced excitonic pairs. Hence, the suppression in excitonic charge pairs in MgCr-LDH/NP is associated with electron and hole trapping sites, which increases the fate of electronic charge pairs for trigging superior water splitting performances. Generally, the smaller the impedance arc radius, the faster the charge carriers separation. The radius corresponding to the above sample Nyquist circle is MgCr-LDH/PS > MgCr-LDH/NS > MgCr-LDH/NP. In summary, the MgCr-LDH/NP combination can not only use the internal oxygen vacancy and Cr^3+^ dopant as barrior for the electron–hole recombination to accelerate the separation of carriers, but also build an effective electron transfer channel, accelerate electron transfer, and improve the charge trapping ability.

In general, photocurrent response is used to reveal the phenomenon of photogenerated electrons generated by photoexcitation of photocatalyst. As we all know, the higher the photocurrent response value, the higher the excitation rate of photo-induced exciton pairs, and minimize the electrons and holes recombination rate. The transient photocurrent responses of three working electrodes under visible light exposure are revealed in Fig. 6d. It could be identified that after structural transformation into MgCr-LDH/NP, the catalyst formed successfully constitutes a dense of nanosheets containing oxygen related defect sites, and the MgCr-LDH/NP working electrode shows a significant increase in photocurrent density. An internal interface is formed within the nanoparticles structure where oxygen vacancies and Cr^3+^ involved in the multi electron process for effective trapping of the electrons separating out from the photogenerated holes for superior water splitting reactions. Moreover, the mesoporosity nature of the as-synthesized MgCr-LDH/NP materials offers high surface area plus more surface active sites for photoelectrochemical reactions to enhance the water splitting performance.

In order to further analyze the electron transfer within the catalysts, Mott-Schottky curves and UV-DRS plots were correlated to calculate the CB and VB edges, respectively. The flat band (E_fb_) of n-type semiconductor is close to the conduction band^17,21^. Therefore, the E_CB_ of MgCr-LDH/PS, MgCr-LDH/NS, and MgCr-LDH/NP could be calculated in RHE as − 0.01 V, − 0.06 V, and − 0.20 V, respectively (*E*RHE = *E*Ag/AgCl + 0.197 + 0.059 pH). Hence, according to UV–Vis spectrum and Mott-Schottky curve, the valence band (E_VB_) of MgCr-LDH/PS, MgCr-LDH/NS, and MgCr-LDH/NP could be calculated as + 2.19 V, + 2.14 V, and + 2. 0 V as E_VB_ = E_CB_ + E_g_, respectively. The test result showed that the flat band potential of MgCr-LDH/NP was negatively shifted in comparison to MgCr-LDH/PS (− 0.01 → − 0.20 V), indicating the upward movement of energy level of n-type MgCr-LDH/NP. Generally, the Fermi level is implicit at 0.1 eV down the CB of n-type semiconductors as like MgCr-LDH/NP (E_f_ = − 0.10 V); alternatively the Ov always occupies a space about 0.9 V depth than the CB of any semiconductor, so for MgCr-LDH/NP (+ 1.1 V)^45^. The XPS and PL spectra also verify the presence of defect site and oxygen vacancies. Moreover, Cr^3+^ cations present electronic arrangement (t^3^_2g_e^0^_g_), which induces charge transfer, separation and electronic capture for facilitating the H_2_ production. These features provide strong support that the upward shifting of energy level is related with the successful formation of nanoparticles (verified from TEM and FESEM results) with defect sites as oxygen vacancies and Cr^3+^ as dopant for triggering excitonic separation.

**Figure 14:**
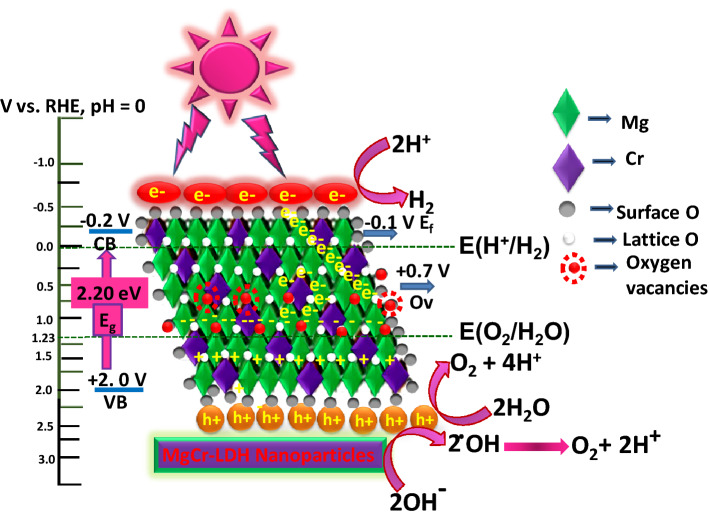
Relative band positions and charge transfer mechanism of the MgCr-LDH/NP for water splitting reaction under visible light exposure.

With these valid discussions, the possible CB and VB position of MgCr-LDH/NP and the mechanism of water reduction and oxidation reaction over MgCr-LDH/NP were proposed in Fig. 14. With the visible light irradiation, semiconductors could absorbed photon energy equal to or greater than the band gap energy, and get excited to produce electrons and hole pairs. The photogenerated electrons transition from the VB position of MgCr-LDH/NP to the CB, and leaving behind holes in the VB. The electrons accumulated on the CB of MgCr-LDH/NP are easily trapped by the Ov center together with the Cr^3+^ cations presents unique electronic arrangement (t^3^_2g_e^0^_g_), which facilitate electron capture to reduce the H^+^ in the solution to facilitate H_2_ production (H^+^/H_2_ (0 vs. RHE), whereas the holes are consumed by the sacrificial agents^45^. Alternatively, rich oxygen vacancies produced on MgCr-LDH/NP containing nanosheets assist simplistic adsorption of water oxidation intermediates such as –OH and –OOH onto the nearby interface of Cr^3+^ and low-coordinated Mg^2+^ ions, which is formed by (i) H_2_O → H + OH, OH + H_2_O → OOH + 2H, and (ii) H_2_O + OH + H → HOOH + 2H^54^. The V_O_ percentage of MgCr-LDH/NP was higher than MgCr-LDH/NS as verified from the peak area fitting in the XPS spectra. Moreover, the LSV curve is also in agreement of defect sites for high current density. Furthermore, water oxidation intermediates are more favorably adsorbed on oxygen vacancies with the help of doped Cr^3+^ in pulling up their electrons. The corresponding Tafel slope is 82 mV/decade and these results confirm that the incorporation of Cr^3+^ is the crucial factor in increasing the reaction kinetics of MgCr-LDH/NP. Cr^3+^ as Lewis acid cations can modulate the ligand fields of the hydroxyl groups of LDH layers. In this way the electrons are concentrated in the CB and attracted towards V_0_ of Cr^3+^ cations and thereafter in the CB of MgCr-LDH/NP and holes intense at VB of MgCr-LDH/NP (+ 2.0 V *vs*. RHE) possess sufficient potential to produce ·OH radicals E^Ѳ^ (·OH/OH– = + 1.99 eV vs. NHE)^17^. Therefore, the holes on the VB of NiFe LDH could react with H_2_O to produce the ·OH radicals, which used for water oxidation reactions (O_2_/H_2_O (1.23 V vs. RHE). Moreover, the hole enriched VB of MgCr-LDH (+ 2.0 V vs. NHE) could directly oxidized H_2_O to produce O_2_ gas. Consequently, both rich oxygen vacancies and doped Cr^3+^ cations lead to the increased charge carrier density and decreased the resistance arises owing to the presence of Mg(OH)_2_ at the interface for charge transfer, thus promoting the kinetics for water splitting reactions. A state of art for comparing the photocatalytic activities of MgCr-LDH/NP and PEC properties with literature reported materials were depicted in Supplementary Tables S2 and S3 in the supporting information, respectively.

**Figure 15:**
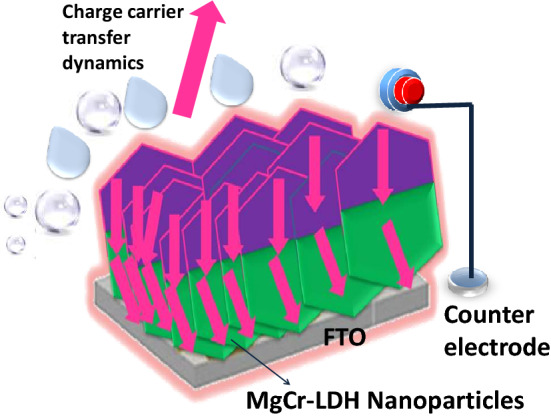
Schematic illustration of the future proposed expected carrier charge transfer dynamics of deposited MgCr-LDH/NP photocatalyst to current-collector substrates.

This type of work exemplify the inherent performance of the photocatalysts by designing the appropriate catalyst composition (Cr^3+^ cation) with defect sites and the effect of high active sites in open framework-3D nanostructures for enhanced PEC properties triggering superior water splitting performance. After constructing the open nanoparticles structure, the contact position of MgCr-LDH/NP can be considered as a small photoelectrochemical (PEC) cell. From the perspective of a PEC photoanode material, the band structures of MgCr-LDH/NP could be best fitted and compared with MgCr-LDH/NS and MgCr-LDH/PS as depicted earlier by comparing UV-DRS and Mott-Schottky plot. The upward shift of CB edge of the MgCr-LDH/NP system offers adequate cathodic potential for H_2_ reduction from H_2_O, causing the water oxidation reaction under visible light exposure as shown in Fig. 15. Hence, the entire MgCr-LDH/NP can be regarded as a high-efficiency PEC cell assembly connected in three electrode series. This is advantageous to the improvement of hydrogen evolution performance. In addition, compared with other variant of LDH-based photoelectrode, the MgCr-LDH/NP photoelectrode also reveals comparable PEC properties, as shown in Supplementary Table S4.

## Conclusions

In summary, we have successfully designed defect-rich 3D nanoparticles-like MgCr-LDHs composed of 2D nanosheets by using a facile hydrothermal and light irradiation method, and taken advantage of these special 3D nanoparticles-like structures that provided added active sites, thereby behave as an effective photocatalysts by reducing the recombination of photo-induced e− and h+ pairs, for enhancing the water splitting activities. In addition, XPS and PL analyses shows the dominance of oxygen vacancies and defects site with special electronic configuration of Cr^3+^ dopant (t^3^_2g_e^0^_g_), and synergistically facilitates charge transfer, conductivity, electron capture and adsorption of water oxidation intermediates for facilitating the H_2_ and O_2_ production. Moreover, the MgCr-LDHs nanoparticles delivered interesting PEC properties with low Tafel slope values of 82 mV/decade for a current density of 6.9 mA/cm^2^, which is significant and these LDH might be used as a potent photoanode material for future PEC water splitting activities. Evidently, MgCr-LDH nanoparticles exhibited superior photocatalytic H_2_ evolution activities of 1315 μmol/h, which was 1.8 and 4.3 times than MgCr-LDH nanosheets (726 μmol/h) and pristine MgCr-LDH (300 μmol/h) under visible-light exposure. Alternatively, MgCr-LDH nanoparticles shows the highest O_2_ production activities of 579 μmol/h, which is 1.6 and 2.2 times superior than MgCr-LDH nanosheets (356 μmol/h) and pristine MgCr-LDH (254 μmol/h), respectively. Additionally, the MgCr-LDH nanoparticles system showed robust recyclability without degradation of their surfaces. Due to the synergistic effects of oxygen vacancies and Cr^3+^ doping, we expect further that nanoparticles derived from LDH nanosheets by hydrothermal and light irradiation could be utilized to attain high activity and robust stability in water splitting even for many other nanostructures. Such eco-friendly binary LDHs can be used as photoanode material in PEC cell for industrial transformation.

## Experimental section

### Materials and methods

Mg(NO_3_)_2_·6H_2_O (98%, Sigma-Aldrich), Cr(NO_3_)_3_·9H_2_O (98%, Sigma-Aldrich), 23 vol% formamide (Sigma-Aldrich), anhydrous NaOH (98%, Sigma-Aldrich), were straight in use for the fabrication method. Nafion solutions were obtained from Sigma-Aldrich-India. Deionized (DI) water was used all through the experimental procedure.

### Strategic fabrication process of pristine MgCr-LDH (Mg:Cr = 3:1)

A simplistic one-pot co-precipitation method was implemented for the synthesis of pristine MgCr-LDH at room temperature by slowly bubbling N_2_ gas throughout the experimental process. At first instance, 10 mL of the DI H_2_O was constantly purged with N_2_ for a minimum period of 15 min. Then 20 mL solution of mixed metal nitrate containing Mg (NO_3_)_2_·6H_2_O (0.030 M) and Cr (NO_3_)_3_·9H_2_O (0.010 M), were drop wise added to the 20 mL of DI H_2_O and 20 mL ethanol under constant N_2_ purging and slow aging at room temperature. After then 1 M NaOH solution was dropwise added into the aqueous solution slowly until the pH of the solution was maintained at about 7 and the resultant suspension was kept under constant aging at room temperature for over 18 h. Consequently, the product isolation was settled by speedy centrifugation of 7000 rotation per minutes (RPM), and finally washed with DI water and ethanol for three times, and then vacum dried at 80 °C overnight. The as-synthesized product was distinct as pristine MgCr-LDH.

### Strategic fabrication process of exfoliated 2D MgCr-LDH (Mg:Cr = 3:1) Nanosheets

In a unusual synthetic protocol, 20 mL of mixed metal nitrate solution containing Mg (NO_3_)_2_·6H_2_O (0.030 M) and Cr (NO_3_)_3_·9H_2_O (0.010 M) were drop wise added to 10 mL solution of 23 vol% formamide with 20 mL of ethanol and constantly stirred to obtain a clear homogenous solution under N_2_ atmosphere at room temperature. Subsequently, the pH adjustment of the mixed metal nitrate solution was constant at pH = 7 by slow addition of aqueous 1 M NaOH solution till the completion of the saturation point of the green precipitate of MgCr-LDH nanosheets. Then the centrifuged MgCr-LDH colloidal gel was washed with deionized water and ethanol 3–4 times and finally dried in vacuum at 40 °C for couple of days.

### Strategic fabrication process of hierarchal 3D MgCr-LDH nanoparticles (Mg:Cr = 3:1)

In order to synthesized hierarchal nanoparticles like 3D MgCr-LDH, the recovered gel of 2D MgCr-LDH nanosheets was again redispersed and diffused in 10 mL of formamide solution and sonicated for 30 min followed by stirring for 30 min under N_2_ atmosphere at room temperature. The formamide treated colloidal dispersion was transferred to 100 mL Teflon lined autoclave reactor and treated at 80 °C for 24 h. After cooling down to room temperature, the as-synthesized MgCr-LDH gel precipitate was slowly aged under the irradiation of visible light for 30 min. Finally, the as-synthesized hierarchal 3D MgCr-LDH was washed with DI water and ethanol for three times and vacum dried overnight at 40 °C for further use. The most significant feature of this proposed synthetic approach is the tremendous simplification and greener route of the synthesis procedure for the nanoparticles like 3D MgCr-LDH composed of 2D nanosheets.

### Photocatalytic water splitting measurement studies

The catalytic competence of the as prepared MgCr-LDH samples were tested towards water splitting reaction under visible light exposure from 125 W Xe lamp (power density = 100 mW cm^−2^) attached to a quartz reactor fitted with Julabo based chiller and 1 M NaNO_2_ as UV cut off filter to filter out visible light of λ ≥ 400 nm. The water splitting reaction was begin with the addition of 0.02 g of catalyst to 20 mL of 10 vol% CH_3_OH solution and other sacrificial agents then purged with N_2_ gas for 15 min to remove any dissolved O_2_ gas to make the environment inert prior to light exposure. Then the reaction suspension was stirred continuously for 1 h to avoid any catalyst settlement under the exposure of visible light. The evolved gas was collected using downward displacement of water and further detected by GC-17A and column packed with 5 Å molecular sieves, set with thermal conductivity detector (TCD). Similar experiment condition was implemented, for O_2_ evolution, with 0.03 g of catalyst added to 30 mL of 10 vol% of AgNO_3_ and other tested sacrificial agents.

Apparent Conversion Efficiency (ACE) for H_2_ evolution = 

The apparent conversion efficiency (ACE) of the MgCr-LDH/NF photocatalyst producing H_2_ gas of 1315 μmol/h and O_2_ gas of 579 μmol/h by using 125 W Hg lamp as the visible light source positioned 9 cm away from the photocatalytic reactor could be determined by using the below mentioned Eq. (9):

24 $$\text{ACE }(\text{\%}) = \frac{\text{Stored chemical energy }}{\text{Incident photon energy}}\times 100,$$

$${\text{H}}_{2}\text{O }\to {H}_{2}+ \frac{1 }{2 }{\text{O}}_{2}\Delta Hc = 285.8\text{ kJ}/\text{mol},$$

where ΔH_c_ = heat of combustion of hydrogen in kJ/mol, Stored chemical energy = (number of moles of hydrogen produced per second) × ΔH_c_ kJ/mol = 0.3652 × 10^–6^ mol/s × 285.8 × 10^3^ J/mol = 0.1043 J/s or W.

The calculated power density for 250 W Hg lamps as visible light source is approximately 100 mW cm^−2^.

Incident photon energy = power density of the incident visible light × (light exposed spherical surface area of the reaction container) = 100 mW cm^-2^ × π × r^2^ (r = radius of the spherical surface = 1.5 cm)

 = 100 mW cm^−2^ × 3.141 × (1.5 cm)^2^ = 0.7067 W.

Thus, ACE (H_2_ evolution) = 0.0521 W/0.7067 W = 0.1475 = 14.75%

Apparent Conversion Efficiency (ACE) for O_2_ evolution = 

The ACE for O_2_ evolution could be calculated by applying same Eq. (24) as used for H_2_ evolution.

Stored chemical energy = Number of moles of O_2_ produced/sec after reaction completion × ΔH_c_ of O_2_ in kJ/mole = 0.1608 × 10^–6^ × 285.8 × 10^3^ = 0.0459 W.

$\text{Incident photon energy}$ = Intensity of 125 W Xe lamp × Area of spherical surface on which light is irradiated (πr^2^) = 100 mW cm^−2^ × 3.141 × (1.5 cm)^2^ = 0.7067 W.

ACE (O_2_ evolution) = 0.0459 W/ 0.7067 W = 0.0649 = 6.4%.

### Preparation of photoelectrode and PEC measurements

#### Electrode preparation

20 mg of each MgCr-LDH samples were suspended in 1 mL ethanol, plus 1 mL methanol and 20 μL Nafion and sonicated for 20 min. Then the mixture was coated onto fluorine-doped tin oxide (FTO) by drop-casted method in the dimension of 1 × 1 cm^2^. The LDH coated thin films were vacuumed dried at 80 °C for overnight prior to use.

#### PEC measurements

PEC measurements were carried out by potentiostat − galvanostat (IviumStat) terminal at a scan rate of 10 mV s^−1^, with accessories of 300 W Xenon lamps of 100 mW/cm^2^ illumination was maintained as the light source, three-electrode system carrying Pt, Ag/AgCl (3.5 M KCl), and FTO, as counter, reference, and working electrode, respectively. 0.1 M Na_2_SO_4_ solution with tested pH 6.5 was used as the electrolyte. 1 sun distance was maintained between the Xenon lamp and photoelectrode for measuring the PEC properties. The linear sweep voltammetry (LSV) study was deliberate by applied bias within − 1.0 to 1.2 V vs. Ag/AgCl reference electrode at scanning rate of 10 mV s^−1^ under visible light exposure. The MgCr-LDH deposited over FTO containing thin films were used as a working electrode with approximately 0.35 cm^2^ geometric area exposed to the electrolyte solution under light irradiation. The experiments were performed in a conventional three-electrode quartz cell using Ag/AgCl (3.5 M KCl) as a reference electrode, Pt-foil as counter electrode and the MgCr-LDH coated film as the working electrode. Electrochemical impedance spectroscopic (EIS) measurements were performed using a similar experimental setup, of 0.1 M Na_2_SO_4_ solution with frequency response of 5000–30 Hz. Mott-Schottky analysis was carried out at DC potential range of − 0.3 V to + 1.5 V vs. RHE with the AC potential frequency 5 kHz and the amplitude of AC potential at 0.050 V under dark condition in 0.1 M Na_2_SO_4_ solution (pH 6.5). The stability of the material undergoing water splitting reaction was examined through chronoamperometry using the same reaction condition under constant illumination of 100 mW/cm^2^ at an applied potential of 0.5 V for 6000 s. The chopped illumination was achieved by an electronic shutter with light on and off cycle of 30 s at constant potential of 0.5 V and the transient LSV measurements were conducted at scan rate of 10 mV/s to match with the chopped illumination cycles.

### Materials characterization techniques

The phase purity of the as-prepared materials were characterized by XRD, Rigaku Miniflex powder diffractometer) with Cu Kα as radiation source (λ = 1.54 Å, 30 kV, 50 mA). The functional groups associated with the bending and stretching mode of vibration of the materials were specified by JASCO FT-IR-4600, using KBr reference. The exterior surface morphology and structural features of the materials were obtained by FESEM by using ZEISS Sigma 500 VP microscope. The internal structure and morphology of the material was explored under the TEM and HR-TEM analysis by using JEOL 2100. The XPS measurement was taken at an X-ray photoelectron spectrometer (ESCALAB 250XI) with X-ray source as nonmonochromatized Mg Kα and energy of 0.8 eV. The optical absorption measurements were recorded by JASCO-V-750 UV–Vis spectrophotometer. The PL emission spectra were recorded by applying excitation energy of 320 nm using JASCO-FP-8300 spectrophotometer. The surface area of the MgCr-LDH based samples were measured by N_2_ adsorption–desorption Brunauere–Emmett–Teller (BET) measurements using NOVA Quantachrome TouchWin v1 0.22. The pore size distribution and pore volume were obtained by applying the BJH model. PEC measurements of samples were carried out by potentiostat–galvanostat (IviumStat) terminal.

## Supplementary Information

Supplementary Information.
