# Supplementary material for: Superlative photoelectrochemical properties of 3D MgCr-LDH nanoparticles influencing towards photoinduced water splitting reactions
Source: Sci Rep. 2022 Jun 3;12:9264. doi: 10.1038/s41598-022-13457-x (PMC9166737; doi:10.1038/s41598-022-13457-x)
Supplement: Supplementary file 1 — Supplementary Information. [file 41598_2022_13457_MOESM1_ESM.docx]

**Supporting Information**

**Superlative Photoelectrochemical Properties of 3D MgCr-LDH Nanoparticles influencing towards Photoinduced Water Splitting Reactions**

Susanginee Nayak ^a^* and K. M. Parida ^a^*

^a^ Centre for Nano Science and Nano Technology, Institute of Technical Education and

Research, Siksha ‘O’ Anusandhan University, Bhubaneswar-751030, Odisha, India

................................................................

^*^Corresponding author

*Dr. Susanginee Nayak*

*E-mail:* [*susanginee@gmail.com*](mailto:susanginee@gmail.com)

*Prof.* *Kulamani Parida*

*E-mail: kulamaniparida@soauniversity.ac.in & paridakulamani@yahoo.com*

*Tel. No: +91-674-2351777; Fax: +91-674-2350642*

**Figure S1.** (a-b) TEM image of MgCr-LDH/NS.

**Figure S2.** (a-c) Elemental color mapping results of MgCr-LDH/NP; (d) Energy dispersive X-ray spectroscopy (EDX) results of MgCr-LDH/NP sample signifying the homogeneous occurrence of Mg, Cr, and O elements.

**Figure S3.** Normalized FT-IR spectra plot of MgCr-LDH/NS and MgCr-LDH/NP.

**Figure S4.** Considerate results of the XPS survey spectra of MgCr-LDH/NS with MgCr-LDH/NP.

**Figure S5.** Chronoamperometric stability test of MgCr-LDH/NP for 6000 s under visible light exposure.

**Figure S6.** Stability test of MgCr-LDH/NP before and after the photocatalytic H_2_ evolution analyzed through XRD pattern.

**Figure S7.** TEM image of MgCr-LDH/NP after the 4^th^ cycle of photocatalytic H_2_ evolution test.

**Table S1.** Crystal structures, crystal system, space group and unit cell parameter of MgCr-LDH/PS, MgCr-LDH/NS, and MgCr-LDH/NP samples as calculated from the XRD pattern.

| Material Details | Structure | Crystal System | Space Group | d-spacing (003) (Ȧ) | d-spacing (006) (Ȧ) | d-spacing (110) (Ȧ) | Lattice constant (Ȧ) |
| --- | --- | --- | --- | --- | --- | --- | --- |
| MgCr-LDH/PS | Hydrotalcite | Hexagonal | R 3 m | 6.3659 | 2.9007 | 1.6682 | c = 18.250 a = 3.336 |
| MgCr-LDH/NS | Hydrotalcite | Hexagonal | R 3 m | 4.8440 | - | 1.6169 | c = -  a = 3 .233 |
| MgCr-LDH/NP | Hydrotalcite | Hexagonal | R 3 m | 6.8571 | 3.8637 | 1.5267 | c = 21.876 a = 3 .053 |

a = 2d_110_; c = (3d_003_ + 6d_006_)/2.

**Table S2.** State of literature report for comparison of photocatalytic H_2_ production over MgCr-LDH/NP with other reported materials.

| Catalytic system | Source | H_2_ Production | Reference |
| --- | --- | --- | --- |
| MgCr-LDH derived MgO/MgCr_2_O_4_ (MgCr 3:1)  nanocomposite | Hg Lamp (125 W) | 840 (µmol g^-1^2h^-1^) | 12 |
| NiFe-LDH/g-C_3_N_4_ | 125 W  medium pressure Hg lamp (λ > 420 nm) | 1488 µmol g^-1^ h^-1^ | 13 |
| CdS/ZnCr-LDH | Xe Lamp (300 W) | 374 µmol g^-1^ h^-1^ | 14 |
| Ni-ZnCr-LDH | 125W  medium pressure Hg lamp (> 420 nm) | 1915 µmol g^-1^ h^-1^ | 15 |
| NiFe-LDH/N-rGO/g-C_3_N_4_ | 125 W  medium pressure Hg lamp (> 420 nm) | 2508 µmol g^-1^ 2h^-1^ | 16 |
| rGO/LTO/NiFe-LDH | Simulated solar light (AM 1.5, 100mW/cm^2^) | 532 μmol g^-1^ h^-1^ | 17 |
| CoAl-LDH/rGO | 300 W Xe lamp (λ ≥ 420 nm | 1571.84 μmol g^-1^ h^-1^ | 18 |
| MoS_2_/NiFe-LDH | 300 W Xe lamp | 550.9 μmol h^-1^ | 19 |
| MoS_2_/CoAl-LDH | 300 W Xe lamp | 17.1 μmol g^-1^ h^-1^ | 20 |
| CoAl-LDH/NiTiO_3_ | 5 W LED | 594 μmol 5h^-1^ | 21 |
| 2D-C_3_N_4_/NiFe-LDH | 450 W solar simulator (AM1.5) | 3087 μmol g^–1^ h^–1^ | 22 |
| ZnCr-LDH/g-C_3_N_4_ | 300 W Xe lamp (λ > 420 nm) | 186.97 μmol g^–1^ h^–1^ | 23 |
| CoAl-LDH/g-C_3_N_4_ | Solar simulator AM 1.5 attached with 300 W Xe lamp | 680.13 μmol g^–1^ h^–1^ | 24 |
| MgCr-LDH/NP | 125 W Xe lamp (power density = 100 mW cm^−2^) | 1315 μmol/h | Present work |

**Table S3.** State of art for comparison of photocatalytic O_2_ production over MgCr-LDH/NP with other reported materials.

| Catalytic system | Source | H_2_ Production | Reference |
| --- | --- | --- | --- |
| CoFe-LDH | 300 W Xe lamp (>400 nm) | 45 µmolg^-1^ 3h^-1^ | 25 |
| Tb-ZnCr-LDH | 150 W Xe lamp (>420 nm) | 1022 µmol g^-1^ h^-1^ | 26 |
| NiTi-LDH | 300 W Xe lamp (>400 nm) | 2148 µmol g^-1^ h^-1^ | 27 |
| NiFe-LDH/N-rGO/g-C_3_N_4_ | 125 W  medium pressure Hg lamp (> 420 nm) | 1280 µmol g^-1^ 2h^-1^ | 16 |
| NiFe-LDH/g-C_3_N_4_ | 125 W  medium pressure Hg lamp (> 420 nm) | 886 μmol g^-1^ h^-1^ | 13 |
| ZnCr-LDH/rGO | 450 W Xe lamp (λ >420 nm) | 1200  μmol g^-1^ h^-1^ | 28 |
| NiAl-LDH/g-C_3_N_4_/Ag_3_PO_4_ | 250 W quartz tungsten halogen lamp | 4330 μmol g^−1^ h^−1^ | 29 |
| MgCr-LDH/NP | 125 W  medium pressure Hg lamp (> 420 nm) | 579 μmol/h | Present Work |

**Table S4.** Comparison of PEC photocurrents density for various catalysts assembled with LDH.

| Name of Sample | Current density | Reference |
| --- | --- | --- |
| Fe_2_O_3_/rGO/NiFe-LDH | 0.39 mA/cm^2^ vs. RHE | 1 |
| ZnCr-LDH derived mixed metal oxide | 73 µA cm^2^ vs. RHE | *2* |
| Fe_2_O_3_/N-doped NiAl-LDH nanosheets | 1.73 mA/cm^2^ vs. RHE | 3 |
| BiVO_4_/ZnCoFe-LDH | 3.43 mA cm^-2^ vs. RHE | 4 |
| CoMnZn-LDH/BiVO_4_ | 0.61 mA cm^-2^ vs. RHE | 5 |
| Graphene@LDH@BiVO_4_ | 2.13 mA·cm^−2^ vs. RHE | 6 |
| TiO_2_/BiVO_4_/NiFe-LDH | 6.9 µA/cm^2^ vs. RHE | 7 |
| 3D BiVO_4_/NiFe-LDH | 1.21 mA·cm^−2^ vs. RHE | 8 |
| BiVO_4_/rGO/NiFe-LDH | 3.26 mA cm^-2^ vs. RHE | 9 |
| CuTi-LDH@g-C_3_N_4_ | J_ph_ = 10 mA cm^-2^ at 0.65 V vs. Ag/AgCl | 10 |
| PbI_2_/Co-Cd–Fe LDH | J_ph_ = 53.3 mA cm^−2^ at − 1 V vs. Ag/AgCl | 11 |
| MgCr-LDH/NP | 6.9 mA/cm^2^ at 1.10 V vs. RHE | Present Work |

**References**

1. Ning, F. *et al*. TiO_2_/graphene/NiFe-layered double hydroxide nanorod array photoanodes for efficient photoelectrochemical water splitting. *Energy Environ. Sci.* **9**, 2633-2643 (2016).

2. Cho, S. *et al*. An exceptionally facile method to produce layered double hydroxides on a conducting substrate and their application for solar water splitting without an external bias. *Energy Environ. Sci*. **7**, 2301-2307 (2014).

3. Kim, K. H. *et al*. Plasma-mediated fabrication of ultrathin NiAl nanosheets having rich oxygen vacancies and doped nitrogen sites and their utilization for high activity and robust stability in photoelectrochemical water oxidation. *J. Mater. Chem.* *A*. **6**, 23283-23288 (2018).

4. Wen, X., Fan, M., Zhao, Q., Li, J. & Liu, G. Boosting the Photoactivity of BiVO_4_ Photoanodes by a ZnCoFe‐LDH Thin Layer for Water Oxidation. *Chem.: Asian J*. **16**, 4095-4102 (2021).

5. Vo, T. G., Tai, Y. & Chiang, C. Y. Multifunctional ternary hydrotalcite-like nanosheet arrays as an efficient co-catalyst for vastly improved water splitting performance on bismuth vanadate photoanode. *Journal of Catalysis*. **370**, 1-10 (2019).

6. Zhang, X. *et al*. Enhancing photoelectrochemical water oxidation efficiency of BiVO_4_ photoanodes by a hybrid structure of layered double hydroxide and graphene. *Ind. Eng. Chem. Res*. **56**, 10711−10719 (2017).

7. Zhou, W. *et al*. Ultrathin TiO_2_/BiVO_4_ nanosheet heterojunction arrays modified with NiFe-LDH nanoparticles for enhanced photoelectrochemical oxidation of water. *Journal of colloid and interface science*, **549**, 42-49 (2019).

8. Zhu, Y. *et al*. Interface engineering of 3D BiVO_4_/Fe-based layered double hydroxide core/shell nanostructures for boosting photoelectrochemical water oxidation. *J. Mater. Chem.* *A*. **5**, 9952-9959 (2017).

9. Chen, H. *et al*. Identifying dual functions of rGO in a BiVO_4_/rGO/NiFe-layered double hydroxide photoanode for efficient photoelectrochemical water splitting. *J. Mater. Chem.* *A*. **8**, 13231-13240 (2020).

10. S. Guru, S. Kumar, S. Bellamkonda, R. R. Gangavarapu, Synthesis of CuTi-LDH supported on g-C_3_N_4_ for electrochemical and photoelectrochemical oxygen evolution reactions. *Int. J. Hydrog. Energy*. **46**, 16414-16430 (2021)

11. F. Mohamed, N. Bhnsawy, M. Shaban. Reusability and stability of a novel ternary (Co–Cd–Fe)-LDH/PbI_2_ photoelectrocatalytst for solar hydrogen production. *Sci. Rep*. **11**, 1-14 (2021).

12. Nayak, S., Pradhan, A. C. & Parida. K. M. Topotactic transformation of solvated MgCr-LDH nanosheets to highly efficient porous MgO/MgCr_2_O_4_ nanocomposite for photocatalytic H_2_ evolution. *Inorg. Chem*. **57**, 8646-8661(2018).

13. Nayak, S., Mohapatra, L. & Parida, K. M. Visible light-driven novel g-C_3_N_4_/NiFe-LDH composite photocatalyst with enhanced photocatalytic activity towards water oxidation and reduction reaction. *J. Mater. Chem. A*. **3**, 18622-18635 (2015).

14. Zhang, G. *et al*. Highly efficient photocatalytic hydrogen generation by incorporating CdS into ZnCr-layered double hydroxide interlayer. *RSC Adv*. **5**, 5823−5829 (2015).

15. Baliarsingh, N., Mohapatra, L. & Parida, K. Design and development of a visible light harvesting Ni–Zn/Cr–CO_3_^2−^ LDH system for hydrogen evolution. *J. Mater. Chem.* *A*. **1**, 4236-4243 (2013).

16. Nayak, S. & Parida, K. M. Deciphering Z-scheme charge transfer dynamics in heterostructure NiFe-LDH/N-rGO/g-C_3_N_4_ nanocomposite for photocatalytic pollutant removal and water splitting reactions*.* *Sci. Rep*. **9***,* 2458-2481 (2019).

17. Boppella, R., Choi, C. H., Moon, J. & Kim. D. H. Spatial charge separation on strongly coupled 2D-hybrid of rGO/La_2_Ti_2_O_7_/NiFe-LDH heterostructures for highly efficient noble metal free photocatalytic hydrogen generation. *Appl. Catal. B Environ*. **239**, 178-186 (2018).

18. Kumaresan, A. *et al*. Facile development of CoAl-LDHs/RGO nanocomposites as photocatalysts for efficient hydrogen generation from water splitting under visible-light irradiation. *Inorg. Chem. Front*. **6**, 1753-1760 (2019).

19. Nayak, S., Swain, G. & Parida, K. Enhanced photocatalytic activities of RhB degradation and H_2_ evolution from in situ formation of the electrostatic heterostructure MoS_2_/NiFe LDH nanocomposite through the Z-Scheme mechanism via p–n heterojunctions. *ACS Appl. Mater. Interfaces.* **11**, 20923-20942 (2019).

20. Tao, J., Yu, X., Liu, Q., Liu, G. & Tang, H. Internal electric field induced S–scheme heterojunction MoS_2_/CoAl LDH for enhanced photocatalytic hydrogen evolution. *J. Colloid Interface Sci*. **585**, 470-479 (2021).

21. Li, Y., Wang, G., Wang, Y. & Jin, Z. Phosphating 2D CoAl LDH anchored on 3D self-assembled NiTiO_3_ hollow rods for efficient hydrogen evolution. *Catal. Sci. Technol*., **10**, 2931-2947 (2020).

22. Yan, J., Zhang, X., Zheng, W. & Lee, L. Y. S. Interface engineering of a 2D-C₃N₄/NiFe-LDH heterostructure for highly efficient photocatalytic hydrogen evolution. *ACS Appl. Mater. Interfaces*. **13**, 24723-24733 (2021).

23. Luo, B., Song, R. & Jing, D. ZnCr LDH nanosheets modified graphitic carbon nitride for enhanced photocatalytic hydrogen production. *Int. J. Hydrog. Energy*. **42**, 23427-23436 (2017).

24. Zhang, J. *et al*. g-C_3_N_4_/CoAl-LDH 2D/2D hybrid heterojunction for boosting photocatalytic hydrogen evolution. *Int. J. Hydrog. Energy*. **45**, 21331-21340 (2020).

25. Kim, S. J., Lee, Y., Lee, D. K., Lee, J. W. & Kang, J. K. Efficient Co–Fe layered double hydroxide photocatalysts for water oxidation under visible light.  *J. Mater. Chem. A.* **2**, 4136-

4139 (2014).

26. Fu, Y. *et al*. Terbium doped ZnCr-layered double hydroxides with largely enhanced visible light photocatalytic performance. *J. Mater. Chem. A*. **4**, 3907-3913 (2016).

27. Y. Zhao. *et al*. NiTi-layered double hydroxides nanosheets as efficient photocatalysts for oxygen evolution from water using visible light. *Chem. Sci*. **5**, 951-958 (2014).

28. Gunjakar, J. L., Kim, I. Y., Lee, J. M., Lee, N. –S. & Hwang, S. –J. Self-assembly of layered double hydroxide 2D nanoplates with graphene nanosheets: an effective way to improve the photocatalytic activity of 2D nanostructured materials for visible light-induced O_2_ generation. *Energy Environ. Sci*. **6**, 1008–1017 (2013).

29. Megala, S. *et al*. The construction of a dual direct Z-scheme NiAl LDH/gC_3_N_4_/Ag_3_PO_4_ nanocomposite for enhanced photocatalytic oxygen and hydrogen evolution. *Nanoscale Adv*. **3**, 2075-2088 (2021).
